# Water-Use Efficiency and Mineral Nutrition of Diverse Legume Species Nodulated by Different Native Rhizobial Isolates: Do Rhizobia Have a Say in the Mineral Nutrition of Their Host Plants?

**DOI:** 10.3390/plants15101478

**Published:** 2026-05-12

**Authors:** Lebogang J. Msiza, Titus Y. Ngmenzuma, Mustapha Mohammed, Felix D. Dakora

**Affiliations:** 1 Department of Crop Sciences, Tshwane University of Technology, Private Bag X680, Pretoria 0001, South Africa; zuma.titus@yahoo.com; 2 Department of Crop Science, University for Development Studies, Tamale P.O. Box TL 1882, Ghana; mmustapha@uds.edu.gh; 3 Department of Chemistry, Tshwane University of Technology, Private Bag X680, Pretoria 0001, South Africa

**Keywords:** legumes, water-use efficiency, photosynthetic rate, mineral nutrients, rhizobial isolates

## Abstract

The benefits of legume-nitrogen-fixing bacteria symbioses are vital in agricultural systems globally. Cross-infectivity studies are important for identifying rhizobial strains with potential for use as inoculants. The native rhizobial isolates inoculated on different legume species are the first step to determining host range and ecological adaptive traits. This study reports on the water-use efficiency and mineral nutrition of diverse legume species cross-inoculated by native rhizobial isolates from Eswatini, Ghana and South Africa under glasshouse conditions. A portable infrared red gas analyzer was used for water use efficiency. Data from a gas exchange study shows that rhizobial strains can significantly influence the photosynthetic functioning of their host plants. As a result, photosynthetic rates differed depending on bacterial compatibility with the host plant, as well as its symbiotic efficacy. Isolate TUTGmGH2 induced greater accumulation of P, K, Mg, Zn, Cu and Mn in soybean and Winged bean, clearly suggesting that rhizobia do have an influence on the mineral nutrition of their host plants. Therefore, these findings further show that native rhizobial isolates can be manipulated to enhance mineral nutrient uptake, promote growth and development and also produce nutrient-dense food with a low environmental impact globally since rhizobia do have an influence on the mineral nutrition of their host plants.

## 1. Introduction

Photosynthesis is the most important physiological process in plants and the carbon cycle of the ecosystem [1,2,3] as it determines the survival and growth of plant species [4,5]. The water-use efficiency (WUE) of plants describes the intrinsic trade-off between carbon fixation and water loss through transpiration, which occurs in dryland species due to evaporation of water from the interstitial tissues of leaves whenever stomata open for CO_2_ acquisition. Water-use efficiency is a key trait that relates carbon to water as the plant’s long-term stable functional characteristic [6]. For plants to function efficiently, gas exchange must be balanced inside and outside the leaf to maximize CO_2_ uptake for photosynthetic carbon assimilation and also to minimize water loss through transpiration [7,8,9]. However, photosynthesis is a sensitive physiological process linked to plant metabolism and development that easily responds to changes in the environment [10,11,12]. At the whole-plant level, WUE is defined as plant dry matter produced per unit of water lost via transpiration [13,14], and is measured from leaf photosynthesis and transpiration, whole-plant functioning on the assumption that they reflect the integrated balance between carbon assimilation and water loss across the entire plant over time. Adams et al. [15] found a strong positive correlation between water use-efficiency and δ^13^C values by N_2_-fixing plants. Kirizii et al. [16] also reported a positive relationship between nitrogen fixation and photosynthesis by alfalfa plants. Scherer et al. [17] also found a positive correlation between leaf area and acetylene reduction and between photosynthetic rate and nitrogen content in a nodulated *Alnus glutinosa* species. Despite the significant role played by nitrogen for plant growth and development, it is the most limiting element among the essential macronutrients needed by plants for growth and physiological functioning [18].

Plants can take up varying amounts of about 90 mineral elements; only 17 of them are essential for plant growth and development [19,20,21]. Essential nutrients are divided into two groups based on the quantity required by plants. The elements required in large quantities are classified as macronutrients, and those in small amounts as micronutrients [22,23,24]. Plant species differ in their ability to accumulate nutrient elements [25,26]. For instance, hyperaccumulators such as Pteris vittata (arsenic) and Alyssum murale (nickel) selectively absorb and store high concentrations of specific elements, while staple crops like maize and wheat often prioritize macronutrients such as nitrogen, phosphorus, and potassium. Among the 17 essential elements for plant growth, macronutrients (e.g., nitrogen, phosphorus, potassium) are required in large quantities, whereas micronutrients (e.g., iron, zinc, manganese) are needed in trace amounts but are equally critical for physiological functions.

Symbiotic nitrogen N nutrition is one way of reducing the application of chemical fertilizers to crop species, especially legumes [27,28]. As the symbiotic partners of legumes, rhizobia not only reduce N_2_ to NH_3_, but they also act as plant growth promoters, as well as enhancers of mineral nutrition in nodulated legumes [29,30,31]. It has been shown earlier that high N_2_-fixing symbiosis uptake and assimilates more mineral nutrients than their low-fixing counterparts [31], and that this symbiotic trait is strain-dependent. In essence, high N_2_-fixing rhizobia are generally more likely to enhance the accumulation of nutrient elements in plant organs than low N_2_-fixing rhizobia. Thus, the symbiotic relationship between legumes and rhizobia has the potential for phytostabilization of hostile environments through the legume/rhizobia symbiosis [32,33,34,35]. Symbiotic legumes can therefore be used to protect soils, while enriching ecosystems with N, supplying land cover, restoring soil function and increasing the diversity of flora and fauna [32,36].

Inoculating legumes with rhizobia has both ecological and economic relevance [34,37], especially beneficial soil microorganisms that deliver ecosystem services through biological N_2_ fixation and have been recognized for their role in phytotechnologies. The ability of soil microbes such as *Rhizobium*, *Bradyrhizobium*, *Bacillus* and *Psedomonas* to improve the nutritional status of plants has been reported [34,38], and the strong relationship between N_2_ fixation and mineral accumulation in legume organs has been documented [31]. The aim of this study was to evaluate leaf photosynthetic rate and water-use efficiency of cross-infected legume species inoculated with rhizobia under glasshouse conditions during the 2021 and 2022 cropping seasons, and to further assess the mineral nutrient composition in the organs of diverse legume species treated with rhizobial isolates from African soils. The findings are also expected to provide further insight into the influence of rhizobial strains, legume species, and their interactions on the accumulation of nutrient elements in their host plants. However, the question that remains to be answered is whether symbiotic rhizobia has a say in the mineral nutrition of their host plants.

## 2. Results

### 2.1. A, gs, E and WUE of Legume Species

#### 2.1.1. A, gs, E and WUE of Cowpea

With cowpea cv. IT10K-817-3 was used as a homologous host in 2021 and cv. IT10K-866-1 in 2022. With cowpea, there were significant differences among the rhizobial isolates tested on the two cowpea cultivars (Tables S1 and S2). With genotype IT10K-817-3, isolate TUTVuSA1, TUTVuSA2, TUTVuSA3, TUTPvES3, TUTGmGH1, TUTGmGH3 and nitrate-fed plants induced markedly greater photosynthetic activity than the other test isolates (Table 1). Stomatal conductance was however higher with isolates TUTPvES1, TUTVsES2, TUTMgSA3 and TUTMgSA1, and lowest with TUTPvES2, TUTPvES3 and TUTGmGH2 (Table S1). Isolates TUTMgSA1, TUTMgSA2, TUTMgSA3, TUTVsES2, TUTVsES3 and TUTPvES1, together with NO_3_-fed and commercial inoculant strain, showed greater leaf transpiration and water loss. Isolates TUTPvES2, TUTPvES3, TUTVuSA1, TUTVuSA2, TUTVsES1 and TUTGmGH3 showed much lower transpiration rate. Isolates TUTPvES2 and TUTPvES3 were associated with greater WUE (Table S1). With cultivar IT10K-866-1 as the host, isolates TUTMgSA3, TUTPvES3, TUTPvES1, TUTPvES2 and the commercial inoculant strain, as well as nitrate-fed plants, and TUTPvES1 elicited greater photosynthetic rates ranging from (10.01 to 17.19 µmol CO_2_ m^−2^ s^−1^). Stomatal conductance was markedly higher in the uninoculated plants (0.18 mmol m^−2^ s^−1^) and isolate TUTMgSA3 (0.16 mmol m^−2^ s^−1^) and lowest with isolate TUTGmGH1 (0.02 mmol m^−2^ s^−1^). However, leaf transpiration rates were higher in plants nodulated by isolates TUTVuSA3, TUTMgSA1, TUTVuSA2, TUTPvES3. WUE was also higher in plants nodulated by isolate TUTGmGH2 (279.67 mmol CO_2_ m^−1^·H_2_O) and nitrate-fed plants (181.00 mmol CO_2_ m^−1^·H_2_O) (Table 2).

#### 2.1.2. A, gs, E and WUE of Bambara Groundnut

Bambara groundnut used as a homologous host in this study included landrace SSD5 and SSD8. The gas exchange parameters showed significant differences (*p* ≤ 0.01) between and among the rhizobial isolates nodulating Bambara groundnut (Tables S1 and S2). With landrace SSD5, isolates TUTGmGH1, TUTGmGH2 and commercial strain, as well as nitrate-fed plants, revealed greater photosynthetic rates with values ranging from 12.34 to 19.00 µmol CO_2_ m^−2^ s^−1^. However, for stomatal conductance, isolates TUTPvES1, TUTGmGH3, TUTVsES1, TUTVsES2, TUTGmGH1, and TUTGmGH2 recorded much higher values. Isolates TUTPvES3, TUTGmGH1, TUTGmGH2 and TUTVsE3 recorded much lower transpiration rates in contrast to TUTVsES1 and TUTVsES2, which exhibited lower transpiration rates. The commercial inoculant strain (222. 66 mmol CO_2_ m^−1^·H_2_O), followed by isolates TUTGmGH1 (172.76 mmol CO_2_ m^−1^·H_2_O), TUTMgSA3 (144.85 mmol CO_2_ m^−1^·H_2_O) and nitrate-fed plants (197.37 mmol CO_2_ m^−1^·H_2_O) induced much greater WUE (Table 1). With Bambara groundnut SSD8, photosynthetic rates ranged from 5.52 µmol CO_2_ m^−2^ s^−1^ (TUTPvES2) to 13.52 µmol CO_2_ m^−2^ s^−1^ (TUTMgSA2) (Table S2). Stomatal conductance was markedly higher for isolates TUTVsES1 and TUTVsES2 and lowest for isolate TUTGmGH2. Isolates TUTGmGH2, TUTPvES2, TUTGmGH3 and the commercial inoculant strains, as well as the nitrate-fed plants, showed much higher WUE compared to isolates TUTVsES2 and TUTVsES3 (Table 2).

#### 2.1.3. A, gs, E and WUE of Kersting’s Groundnut

Kerting’s groundnut landraces Puffeun and Dowie were used as hosts for the rhizobial isolates. All the gas exchange parameters measured showed a significant difference among the isolates tested on the two landraces (Tables S1 and S2). With genotype Puffeun, isolates TUTGmGH2 and the commercial inoculant strain showed greater photosynthetic rates compared to the other isolates. The stomatal conductance differed among isolates and ranged from 0.09 µmol CO_2_ m^−2^ s^−1^ for TUTVuSA2 and TUTMgSA1 to 0.27 µmol CO_2_ m^−2^ s^−1^ for isolate TUTMgSA3. Leaf transpiration rates were markedly higher with isolates TUTVuSA3 and TUTMgSA1, and lower with isolate TUTMgSA2. WUE was higher with isolate TUTGmGH2 relative to the other isolates (Table 1). With landrace Dowie as host, isolate TUTGmGH2 (16.45 µmol CO_2_ m^−2^ s^−1^) recorded the highest photosynthetic rates, and TUTMgSA1 (6.88 µmol CO_2_ m^−2^ s^−1^) recorded the lowest. Stomatal conductance also rose with root infection by isolates TUTMgSA1, TUTMgSA2, and TUTVuSA3 relative to the other test isolates. Leaf transpiration rate was highest with isolate TUTPvES3 (1.30 mmol m^−2^ s^−1^) and lowest with commercial inoculant (0.14 mmol m^−2^ s^−1^). Isolates TUTPvES3, TUTGmGH3, TUTGmGH2, and TUTMgSA3 elicited greater WUE than the other isolates (Table 2).

#### 2.1.4. A, gs, E and WUE of Common Bean

With the common bean, cv. NUA 734 and cv. NUA 721 were used as hosts in the cross-infectivity test. Photosynthetic rates were highest for nitrate-fed plants (9.41 µmol CO_2_ m^−2^ s^−1^) and lowest for isolate TUTPvES2 (6.35 µmol CO_2_ m^−2^ s^−1^). Stomatal conductance values were also highly significant (*p* ≤ 0.01) and ranged from 0.04 mmol m^−2^ s^−1^ for TUTPvES2 to 0.07 mmol m^−2^ s^−1^ for isolates TUTGmGH2, commercial inoculant, nitrate-fed plants and uninoculated plants. Isolates TUTPvES2 recorded higher WUE compared to the other test isolates (Table 1). With the common bean cv. NUA 721, photosynthetic rates were higher with isolates TUTPvES1, TUTPvES2, TUTPvES3, and the commercial inoculant. Stomatal conductance was highest with isolate TUTPvES2 (0.17 mmol m^−2^ s^−1^) and lowest with TUTGmGH1 (0.03 mmol m^−2^ s^−1^). Transpiration rates were highest with isolate TUTPvES2, TUTGmGH1, and TUTPvES1, leading to greater water loss. Leaf transpiration values ranged from (0.11 to 0.76 mmol m^−2^ s^−1^). Isolate TUTGmGH1 induced greater WUE, followed by the commercial inoculant strain TUTPvES3, and TUTPvES1 (Table 2).

#### 2.1.5. A, gs, E and WUE of Soybean

Soybean cv. TGX1740-2F and cv. TGX1937-1F were used as the host plants for the cross-infectivity assay. With soybean cv. TGX1740-2F, photosynthetic rates were significant among the isolates used for inoculation, and values ranged from 13.87 µmol CO_2_ m^−2^ s^−1^ to 25.52 µmol CO_2_ m^−2^ s^−1^ for uninoculated plants and isolate TUTVsES2, respectively. Stomatal conductance was highest for isolate TUTGmGH1 (0.27 mmol m^−2^ s^−1^) and lowest for isolates TUTVuSA1, TUTVsES2 and uninoculated plants (0.10 mmol m^−2^ s^−1^). Leaf transpiration was highest in nitrate-fed plants, followed by isolates TUTGmGH2 and TUTPvES3. In this study, isolate TUTMgSA3 induced the highest WUE in soybean (Table 1). With cv. TGX1937-1F, isolates TUTGmGH1, TUTGmGH3, TUTMgSA2, and TUTPvES2 induced an increase in photosynthetic rates compared to the other test isolates. Stomatal conductance was, however, markedly increased by isolate TUTPvES3 (0.21 mmol m^−2^ s^−1^) and decreased by isolates TUTVsES1, TUTVuSA3 and TUTGmGH3 (0.04 mmol m^−2^ s^−1^). Leaf transpiration was higher for the nitrate-feeding plants, followed by isolates TUTGmGH2, TUTPvES3, TUTVsES2 and TUTMgSA3. Isolate TUTGmGH3 induced greater WUE, followed by TUTGmGH1 and TUTVsES2, compared to other test isolates (Table 2).

#### 2.1.6. A, gs, E and WUE Winged Bean

With Winged bean, photosynthetic rates differed significantly, with values ranging from 19.10 µmol CO_2_ m^−2^ s^−1^ for TUTPvES3 to 11.24 µmol CO_2_ m^−2^ s^−1^ for TUTGmGH2. The induced levels of stomatal conductance were higher for isolates TUTGmGH2 (0.46 mmol m^−2^ s^−1^), TUTGmGH1 (0.36 mmol m^−2^ s^−1^), and TUTMgSA3 (0.32 mmol m^−2^ s^−1^) and lower for uninoculated plants (0.10 mmol m^−2^ s^−1^). Isolates TUTGmGH3 and TUTPvES3 elicited greater transpiration rates compared to the other isolates. WUE was highly significant (*p* ≤ 0.01) and showed higher values for isolate TUTPvES3 (136. 43 mmol CO_2_ m^−1^.H_2_O) (Table 1).

#### 2.1.7. A, gs, E and WUE of Velvet Bean

Velvet bean cv. IIHR PS1 and cv. IIHR PS2 were used as the host plants for testing cross-infectivity. With Velvet bean cv. IIHR PS1, isolates TUTPvES2, TUTGmGH1, and TUTVuSA1, recorded much higher photosynthetic rates compared to other test isolates (Table 1). With stomatal conductance values ranging from 0.04 to 0.06 mmol m^−2^ s^−1^. Leaf transpiration rates were also higher for isolate TUTPvES3 (2.87 mmol m^−2^ s^−1^) and lower for isolate TUTGmGH1 (1.75 mmol m^−2^ s^−1^). WUE ranged from 99.83 to 249.00 mmol CO_2_ m^−1^·H_2_O (Table 1). With Velvet bean cv. IIHR PS2, isolate TUTVsES2, TUTPvES3, and TUTPvES2 elicited the highest photosynthetic rates. Stomatal conductance was also increased by isolates TUTPvES2 and TUTVuSA3, while leaf transpiration was increased by only isolate TUTPvES2 (3.04 mmol m^−2^ s^−1^). Plant WUE differed significantly and ranged from 51.04 mmol CO_2_ m^−1^·H_2_O for isolate TUTVuSA3 to 222.45 mmol CO_2_ m^−1^·H_2_O for isolate TUTGmGH3 (Table 2).

#### 2.1.8. A, gs, E and WUE of Jack Bean

Jack bean Accessions 493 and 498 were used as the host plants for the cross-infectivity test. With Accession 493, leaf photosynthetic rates ranged from a high 14.56 µmol CO_2_ m^−2^ s^−1^ for TUTMgSA3 to 5.28 µmol CO_2_ m^−2^ s^−1^ for TUTPvES3. Isolate TUTMgSA3 (0.07 mmol m^−2^ s^−1^) elicited the highest stomatal conductance, with the lowest (0.02 mmol m^−2^ s^−1^) being recorded by isolate TUTPvES3. Isolate TUTVsES2 showed a high water loss of 2.89 mmol m^−2^ s^−1^ through leaf transpiration rate, and uninoculated plants showed the lowest of 1.77 mmol m^−2^ s^−1^. Isolates TUTPvES3 elicited greater WUE than the other isolates (Table 1). With cv. Accession 498, leaf photosynthetic rates varied from 12.38 µmol CO_2_ m^−2^ s^−1^ by nitrate-fed plants to 3.55 µmol CO_2_ m^−2^ s^−1^ by TUTVuSA2. Isolate TUTMgSA1 (0.22 mmol m^−2^ s^−1^), followed by TUTPvES3 (0.17 mmol m^−2^ s^−1^) and TUTVsES3 (0.13 mmol m^−2^ s^−1^), showed greater stomatal conductance than the other isolates. Transpiration rates also rose with isolates TUTMgSA3 (1.55 mmol m^−2^ s^−1^), TUTPvES2 (1.44 mmol m^−2^ s^−1^), TUTVuSA2 (1.18 mmol m^−2^ s^−1^) and TUTGmGH2 (1.05 mmol m^−2^ s^−1^). Isolate TUTVuSA1, TUTVuSA3, nitrate-fed plants and TUTVsES1 induced greater WUE, while TUTMgSA1 recorded the lowest (Table 2 and Table S2).

#### 2.1.9. A, gs, E and WUE of Pigeonpea

Pigeonpea cv. ICEAP500557 and cv. ICEAP00850 were used as the host plants for the cross-infectivity test. With cv. ICEAP500557, isolate TUTMgSA3 induced high photosynthetic rates (21.47 µmol CO_2_ m^−2^ s^−1^) while TUTVsES3 recorded the lowest (9.53 µmol CO_2_ m^−2^ s^−1^). In isolate TUTMgSA3, it also elicited greater stomatal conductance, followed by TUTMgSA2, TUTGmGH2, TUTPvES3, TUTPvES2 and TUTVsES2. Isolates TUTMgSA3 (0.40 mmol m^−2^ s^−1^), TUTMgSA2 (0.31 mmol m^−2^ s^−1^), TUTGmGH2 (0.27 mmol m^−2^ s^−1^) and TUTPvES2 (0.24 mmol m^−2^ s^−1^) recorded increased transpiration rates, leading to high water loss. Plant WUE ranged from 45.15 mmol CO_2_ m^−1^·H_2_O for TUTMgSA3 to 177.70 mmol CO_2_ m^−1^·H_2_O for TUTGmGH1 (Table 1 and Table S1). With cv. ICEAP00850, leaf photosynthetic rates ranged from 7.94 µmol CO_2_ m^−2^ s^−1^ for TUTVsES1 to 17. 61 µmol CO_2_ m^−2^ s^−1^ for TUTPvES3. Isolate TUTGmGH1 recorded the highest stomatal conductance (0.20 mmol m^−2^ s^−1^), and TUTVsES1 the lowest (0.05 mmol m^−2^ s^−1^). Leaf transpiration induced by isolate TUTGmGH2 was significantly higher, followed by TUTPvES2, TUTMgSA2, TUTVuSA2 and TUTGmGH3 compared to the other isolates. Plant WUE ranged from 51.22 to 217.30 mmol CO_2_ m^−1^·H_2_O (Table 2 and Table S2).

#### 2.1.10. A, gs, E and WUE of Mungbean

Mungbean cv. VC1973 and cv. VC6153 (B-20p) were used for the cross-infectivity assay with rhizobial isolates. With cv. VC1973 as the host plant, photosynthetic rate was higher for isolate TUTVsES1, followed by TUTVsES2, TUTPvES3 and TUTGmGH2 compared to the other isolates. Stomatal conductance ranged from 0.07mmol m^−2^ s^−1^ for TUTVsES2 to 0.33 mmol m^−2^ s^−1^ for TUTMgSA3. Isolate TUTMgSA3, TUTPvES1 and TUTVuSA2 showed higher leaf transpiration rate relative to the other isolates. WUE values were 55.11 and 150.26 mmol CO_2_ m^−1^.H_2_O, for isolates TUTVuSA2 and TUTGmGH1, respectively (Table 1 and Table S1). With cv. VC6153 (B-20P), isolates TUTVsES2 (18.31 µmol CO_2_ m^−2^ s^−1^), TUTMgSA3 (15.06 µmol CO_2_ m^−2^ s^−1^), TUTVsES3 (14.39 µmol CO_2_ m^−2^ s^−1^) and TUTPvES3 (13.20 µmol CO_2_ m^−2^ s^−1^) recorded high photosynthetic rates while isolate TUTGmGH2 showed the lowest (5.60 µmol CO_2_ m^−2^ s^−1^). Stomatal conductance ranged from 0.03 mmol m^−2^ s^−1^ for TUTGmGH2 to 0.15 mmol m^−2^ s^−1^ for TUTVuSA1. Transpiration rate was also high for isolate TUTPvES1 (1.67 mmol m^−2^ s^−1^), followed by TUTGmGH1 (1.30 mmol m^−2^ s^−1^), TUTMgSA1 (1.23 mmol m^−2^ s^−1^) and TUTVsES3 (0.94 mmol m^−2^ s^−1^). Isolate TUTVsES3 (210.02 mmol CO_2_ m^−1^·H_2_O) elicited the highest WUE, while isolate TUTVuSA1 (61.62 mmol CO_2_ m^−1^·H_2_O) exhibited the lowest (Table 2 and Table S2).

### 2.2. Macronutrient and Micronutrient Concentrations in Shoots of Legume Species

#### 2.2.1. Cowpea (*Vigna unguiculata*)

**Figure 1:**
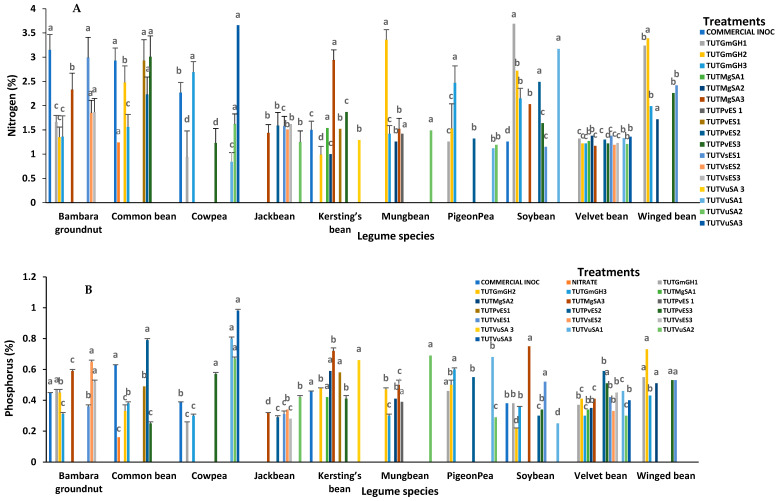

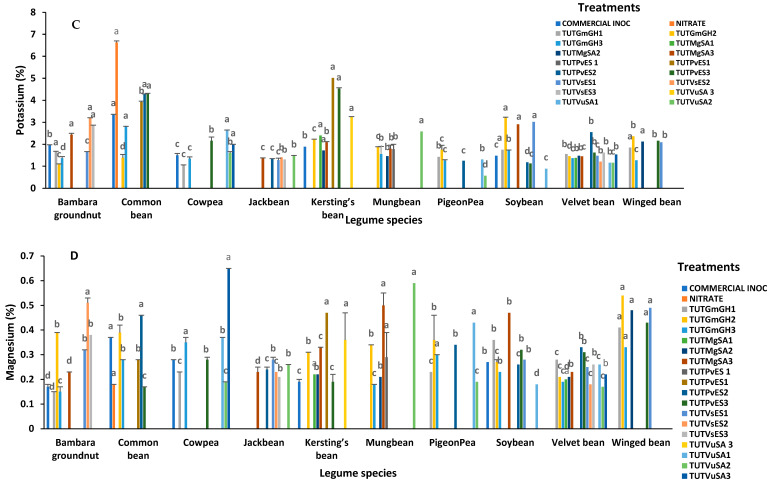

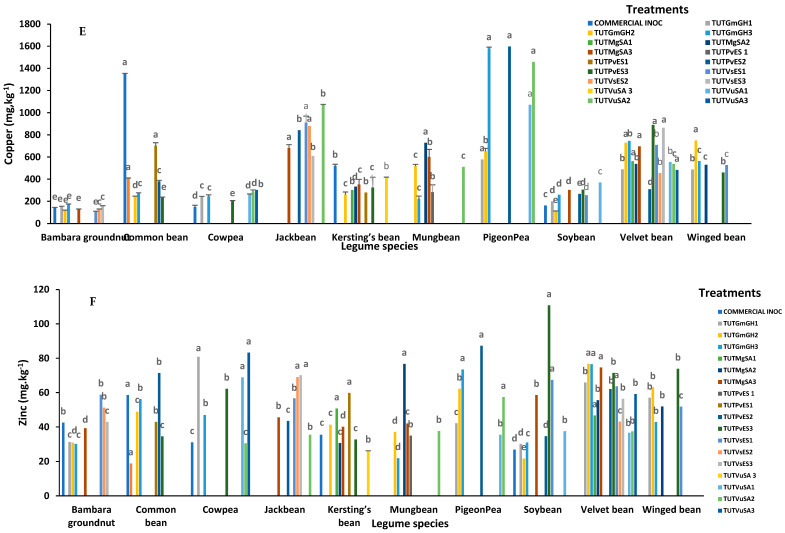

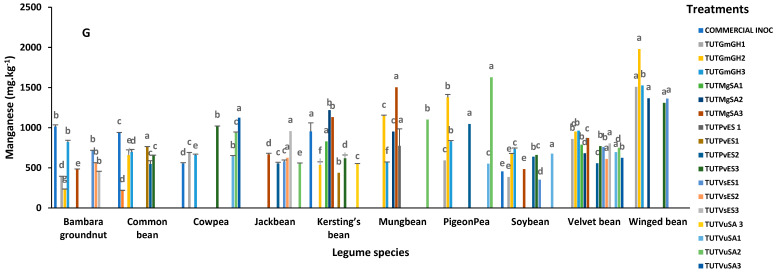
Macronutrient and micronutrient concentrations in shoots of legume species in 2021: (**A**) nitrogen, (**B**) phosphorus, (**C**) potassium, (**D**) magnesium, (**E**) copper, (**F**) zinc, (**G**) manganese. Bars with dissimilar letters are significantly different at *p* < 0.05. Missing bars are denoted as zero nodulation.

**Figure 2:**
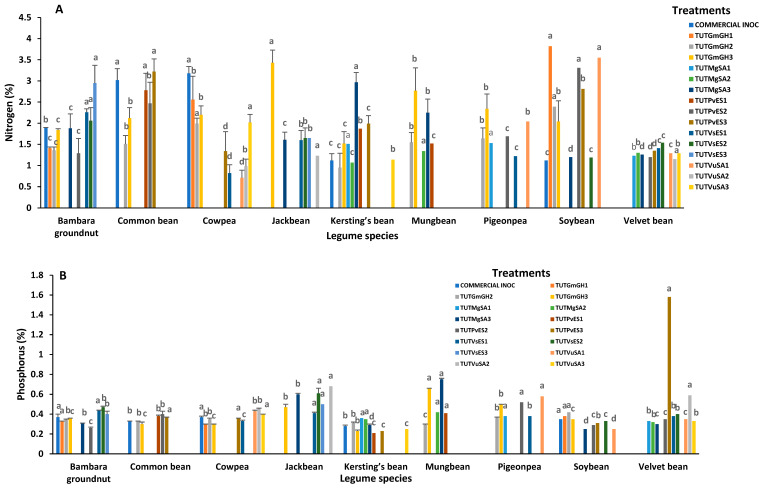

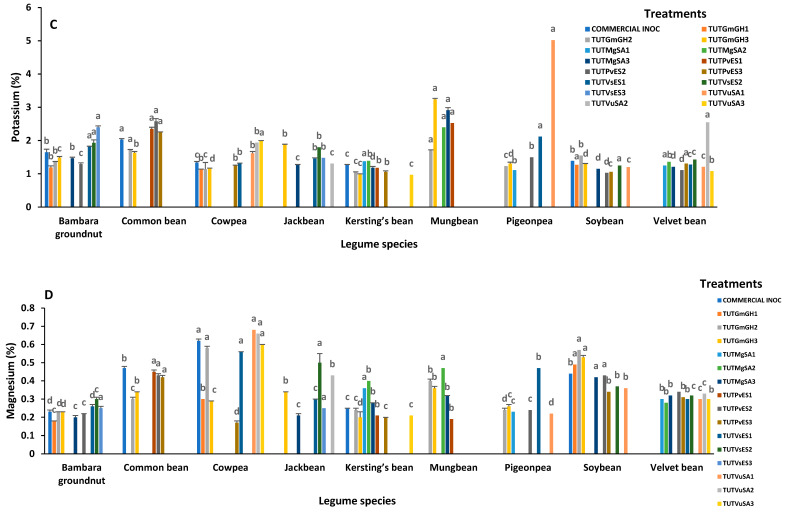

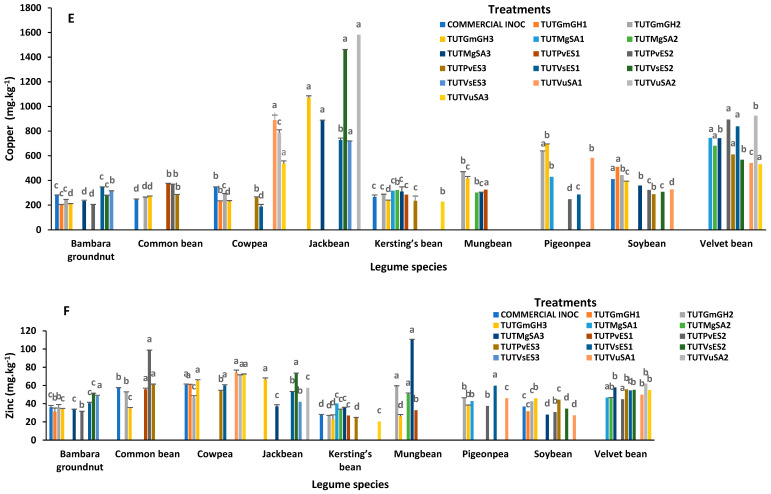

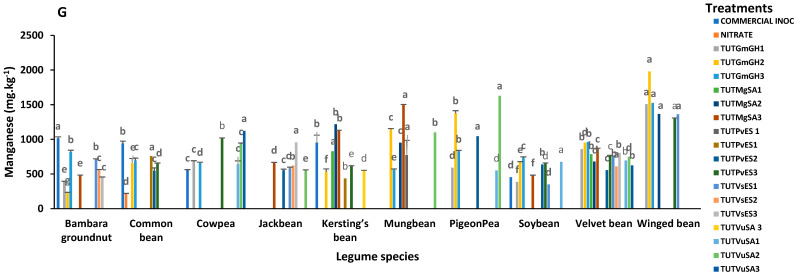
Macronutrient and micronutrient concentrations in shoots of legume species in 2022: (**A**) nitrogen, (**B**) phosphorus, (**C**) potassium, (**D**) magnesium, (**E**) copper, (**F**) zinc, (**G**) manganese. Bars with dissimilar letters are significantly different at *p* < 0.05. Missing bars are denoted as zero nodulation.

The major mineral elements in the shoots of cowpea cv. IT10K-817-3 and cv. IT10K-866-1 (e.g, N, P, K and Mg) differed significantly among the test treatments in 2021 (Tables S3 and S4). Across the board, isolate TUTVuSA3 induced much greater accumulation of N, P, Mg, Cu, Zn, and Mn in shoots than the other isolates (Figure 1A,B,D–G). Isolate TUTVuSA1 recorded the next higher levels of P, K, Mg, and Zn with cowpea cv, IT10K-817-3. Isolate TUTGmGH1 and the commercial strain showed lower accumulation of P, K, Mg, Cu, Zn and Mn. In 2022, TUTVuSA1, TUTVuSA2 and TUTVuSA3, isolated originally from cowpea, recorded a higher shoot level of P, K, Mg, Cu, Zn and Mn (Figure 2B–G).

#### 2.2.2. Bambara Groundnut (*Vigna subterranea*)

With Bambara groundnut landrace SSD5 as the host plant in 2021, shoot macronutrient and micronutrient concentrations varied significantly among the tested rhizobial isolates (Table S3). Isolate TUTVsES2 induced greater shoot accumulation of P, Mg, Cu, K and Zn than the other isolates, followed by TUTVsES1, which also recorded high levels of N and Zn (Figure 1A–E). The results in 2022, with landrace SSD8 as the host plant, showed that isolate TUTVsES2 again recorded the highest levels of P, Mg, Zn, Mn, while TUTVsES1 also induced increased accumulation of N, P, Cu, Mn, Mg and Zn (Figure 2A–F).

#### 2.2.3. Kersting’s Groundnut (*Macrotyloma geocarpum*)

With Kersting’s groundnut landrace puffeun as the host plant in 2021, shoot mineral nutrients varied significantly among the isolates used for inoculation (Table S3). Rhizobial isolate TUTMgSA3 with the highest shoot %N, also elicited greater accumulation of P, Mn, Mg, Cu, and Zn, followed by TUTPvES1 with high levels of K, Mg and Zn (Figure 1A–G). With landrace Dowie as the host plant in 2022, rhizobial isolates TUTMgSA1, TUTMgSA2 and TUTMgSA3 obtained originally from Kersting’s bean accumulated the highest concentrations of mineral nutrients among the other isolates used for inoculation. The three isolates accumulated the highest level of Cu in Kersting’s bean; TUTMgSA1 and TUTMgSA2 also induced the highest levels of P and K in Kersting’s bean (Table S4). As shown in Table S2, strain TUTMgSA1 induced the highest concentration of P, K, Cu, Zn, Mg and Mn, just as TUTMgSA2 increased the levels of P, K, Mg, Cu, and Zn, and TUTMgSA3 raised the levels of N, Cu, Mn, and Zn (Figure 2A–G).

#### 2.2.4. Common Bean (*Phaseolus vulgaris*)

With the common bean cv. NUA 734 as the host plant, isolate TUTPvES2 induced greater accumulation of N, P, K, Mg and Zn in plant shoots in 2021. Isolate TUTGmGH2 also elicited higher accumulation of N and Mg in shoots, while TUTPvES3 markedly increased shoot concentration of N and K in common bean (Figure 1A–F). With cv. NUA721 as the host plant in 2022, the rhizobial isolates obtained originally from common bean accumulated more minerals in cv. NUA721. For example, TUTPvES1 induced greater concentrations of N, P, K, Mg and Cu, while TUTPvES2 exhibited higher levels of P, K, Cu, Zn and Mn. Isolate TUTPvES3 also increased the levels of N, P, Mn and Zn (Figure 2A–G).

#### 2.2.5. Soybean (*Glycine max*)

With soybean cv. TGX1740-2F as the host plant in 2021, isolate TUTVuSA1 induced greater accumulation of N, Cu, and Mn in shoots, while TUTPvES3 caused an increase in Cu, Zn, and Mn in shoots of soybean (Figure 1A,E–G). Isolate TUTMgSA3 similarly increased the concentration of P and Mg in soybean shoots. With soybean cv. TGX1937-1F as the host plant in 2022, rhizobial isolate TUTGmGH2 (obtained originally from soybean) induced higher accumulation of P, K, Mg, Zn, Cu and Mn in soybean shoots. Isolate TUTGmGH1 also caused an increase in the accumulation of N and Cu in shoots (Figure 2A–G).

#### 2.2.6. Winged Bean (*Psophocarpus tetragonolobus*)

Of the six rhizobial isolates used to inoculate Winged bean, TUTGmGH2 stimulated increased accumulation of N, P, K, Mg, Cu, Mn, and Zn in 2021 (Figure 1A–G).

#### 2.2.7. Velvet Bean (*Mucuna pruriens*)

With Velvet bean cv. IIHRPS1 as the host plant, isolate TUTPvES2 stimulated the accumulation of P, K, and Mg in the shoots of Velvet bean in 2021. Shoot N levels were similar for all isolates. With cv. IIHRPS2 as the host plant in 2022, rhizobial strain TUTVuSA2 induced significant accumulation of K, Mg, Cu, Zn and P in shoots of Velvet plants. Isolate TUTPvES2 also stimulated increased accumulation of Mg and Cu in Velvet bean shoots.

#### 2.2.8. Jack Bean (*Canavalia ensiformis*)

With Jack bean Accession 493 as the host plant, shoot N levels were similar in 2021. However, isolate TUTVuSA2 induced a large accumulation of P, K, Cu and Mg in shoots (Table S3). TUTVsES2 also stimulated increased levels of K, Zn, and Cu, while TUTVsES3 caused an increase in shoot levels of Zn and Mn. Isolate TUTVsES1 recorded high levels of Mg, Cu and Zn in shoots. With Accession 498 as the host plant in 2022, isolate TUTVsES2 induced marked accumulation of K, Mg, Zn, Mn, P and Cu in Jack bean shoots. Isolate TUTGmGH3 also caused an increase in shoot levels of P, K, Mn, and Zn in 2022.

#### 2.2.9. Pigeonpea (*Cajanus caja*)

With pigeonpea cv. ICEAP500557 as the host plant, rhizobial isolate TUTPvES2 induced high accumulation of Mg, Cu, Zn, and Mn (Figure 1D–G). Isolate TUTGmGH2 also stimulated the accumulation of K, Mg, and Mn in pigeonpea shoots in 2021. With cv. ICEAP008850, isolate TUTVuSA1 induced high accumulation of N, P, K, Cu and Zn in pigeonpea shoots. Isolate TUTGmGH3 similarly caused an increase in the levels of N, Cu, and P in pigeonpea shoots. Isolate TUTVsES1 similarly induced the accumulation of Mg, Zn, and K in pigeonpea shoots (Figure 2A–G).

#### 2.2.10. Mungbean (*Vigna radiata*)

With mungbean cv. VC1973A as the host plant, isolate TUTVuSA2 elicited the accumulation of P, K, and Mg in mungbean shoots. Isolate TUTMgSA3 also caused an increase in the levels of Mg, Mn, and Cu in leaves, while TUTGmGH2 induced the accumulation of N, K, Mg, Cu, and Mn in mungbean shoots. TUTMgSA2 also stimulated an increase in shoot concentration of Cu and Zn.

## 3. Discussion

### 3.1. A, gs, E and WUE in Shoots of Legume Species

Stomata are the “gatekeepers” responsible for gaseous diffusion, and they adjust to both internal and external environment stimuli, governing CO_2_ uptake and water loss through transpiration [7]. Gas exchange studies involving 11 legume species inoculated with 15 native rhizobial isolates were conducted using two different genotypes of each legume as host plants. The results revealed significant differences in gas exchange parameters associated with the nodulating isolate of the host plant. Furthermore, uninodulated/non-nodulated and ineffectively nodulated plants of the test legume species showed relatively lower photosynthetic rates, stomatal conductance and leaf transpiration, findings consistent with those of an early study by Xiao et al. [39].

Although the photosynthetic rates in 2022 were lower than those of 2021, the values were within the range reported for these species [40]. Photosynthesis is generally affected by the sink strength, described as the photosynthate supply required to meet the increasing demand for photo-assimilates during early vegetative growth and seed development [41]. Furthermore, the photosynthate produced in legumes enhances N_2_ fixation, which is a sink, requiring C for N_2_ reduction. However, fixed N from nodules is exported to leaves as a sink for more Rubisco biosynthesis needed for photosynthesis [42]. N_2_ fixation and photosynthesis are therefore interlinked functionally.

In general, plants with higher stomatal conductance tend to exhibit greater CO_2_ assimilation rates and grow faster under optimal environmental conditions. However, they show lower WUE, as found in this study, with some inoculated plants. It was interesting to note that isolates TUTGmGH2 and TUTGmGH3, which were highly promiscuous and nodulated a wide range of legumes, induced high levels of A, gs and E for increased plant growth. These are the type of rhizobial strains that promote plant growth through increased photosynthetic rates when used as inoculants. However, these promiscuous isolates were not the only ones that induced greater levels of A, gs and E. The gas exchange studies also revealed marked differences in WUE in all the test species inoculated with rhizobia.

Of the 15 rhizobial isolates used in the cross-infectivity assay, three bacterial strains originated from each of five legumes, namely cowpea, Bambara groundnut, Kersting’s groundnut, common bean and soybean. It was interesting to note that in both 2021 and 2022, rhizobial isolates were not necessarily more effective in inducing greater photosynthetic rates in their homologous host. For example, in 2021, Bambara groundnut landrace SSD5, Kersting’s groundnut landrace Puffeun and cowpea cv. IT10K-817-3 recorded greater photosynthetic rates when inoculated with isolates TUTGmGH1, TUTGmGH2 and TUTGmGH3 obtained from soybean. In 2022, the soybean isolates again induced higher photosynthetic rates in Kersting’s groundnut, Velvet bean and their homologous host soybean. Similarly, isolates TUTPvES1, TUTPvES2, and TUTPvES3 from common bean elicited greater photosynthetic rates in cowpea, soybean, Winged bean, Velvet bean, and chickpea in 2021, and again in 2022. About 90% of plant growth and biomass is from C accumulated via photosynthesis. These results therefore indicate the importance of soil microbes in influencing plant growth and development via photosynthesis. It was also interesting to note that there were isolates that induced greater photosynthetic rates, and plant water-use efficiency was also high. For example, in 2021, TUTPvES2, TUTPvES3, TUTGmGH1, TUTGmGH2, TUTGmGH3, and in 2022, TUTPvES2 and TUTPvES3 were among the isolates that elicited high photosynthetic rates and greater WUE in cowpea. The same can be said of TUTVuSA3 and TUTMgSA2 in 2021, as well as TUTMgSA2 and TUTVuSA3 in 2022 for Bambara groundnut; TUTVsES3, TUTPvES1, TUTGmGH1, TUTGmGH2, TUTGmGH3 and commercial strain in 2021, as well as TUTVuSA2, TUTPvES1, TUTPvES2, TUTGmGH1, TUTGmGH2 and TUTGmGH3 in 2022 for Velvet bean; isolate TUTVsES1, TUTVsES2 and TUTVsES3 in 2022 for mungbean.

### 3.2. Macronutrient and Micronutrient Concentrations in Shoots of Legume Species

This study further assessed mineral accumulation by 10 diverse legume species inoculated with native rhizobial isolates in a cross-infectivity test under glasshouse conditions, using two cultivars per test legume, besides the Winged bean, only one cultivar. The cross-nodulation study included cowpea, Bambara groundnut, Kersting’s groundnut, common bean, soybean, Winged bean, Velvet bean, Jack bean, pigeonpea and mungbean. The strains used in this study were originally isolated from root nodules of cowpea, Bambara groundnut, Kersting’s bean, common bean and soybean. The results showed that legume inoculation with rhizobia improved plant growth, mineral nutrient uptake and accumulation in plant parts, including leaves and grain [29]. The increased supply of symbiotic N induced the accumulation of nutrient elements, showing that rhizobia are indeed plant growth promoters capable of reducing the use of chemical fertilizers in agriculture. Given their ability to synthesize protein from symbiotic N and to elicit nutrient uptake and assimilation, there is a growing demand for legumes due to their nutritional benefits for humans, animals, and the environment.

In this study, rhizobial strains that induced nodulation and N_2_ fixation in their homologous host generally promoted an increase in mineral nutrient accumulation in host plant organs (Tables S3 and S4). This was supported by an earlier finding, which showed that rhizobial strains that increased plant growth and biomass also stimulated increased accumulation of nutrient elements in host plant organs [43]. Of the rhizobial isolates tested in the cross-infectivity assay, the majority that caused an increase in tissue nutrient accumulation originated as microsymbionts of cowpea, Bambara groundnut, common bean, soybean and Kersting’s groundnut.

It was interesting to note that isolate TUTPvES2 also effectively nodulated pigeonpea in 2021 and Velvet bean in 2022 and induced the accumulation of Mg, Cu, Zn and Mn in the former and Mg, Cu and Mn in the latter. Isolate TUTPvES1 also nodulated Kersting’s bean and soybean and increased shoot concentration of K, Mg and Zn in the former, as well as Cu, Zn and Mn in the latter. The common bean rhizobia were thus promiscuous in their ability to nodulate Kersting’s groundnut, Velvet bean, soybean and pigeonpea in addition to common bean, the legitimate homologous host. These results are not unexpected as common bean is nodulated by diverse microsymbionts [44]. In 2022, however, all three isolates (TUTMgSA1, TUTMgSA2 and TUTMgSA3) elicited increased concentrations of minerals in host plant shoots. Isolate TUTMgSA1 induced greater shoot accumulation of P, K, Mg, Cu, Zn and Mn; TUTMgSA2 stimulated increased concentration, while TUTMgSA3 caused higher accumulation of N, Cu, Zn and Mn in Kersting’s groundnut, as well as N, P, K and Cu in Mungbean shoots. Although the results obtained in this study are interesting, interpretation remains difficult due to a range of factors. Independent of the effect of bacterial inoculation, growth rates can differ between and among the legume species tested, leading to differences in mineral nutrient demand often linked to source-sink strength. The fact that plants were supplied with a complete nutrient solution except N implies that uptake rates were not limited by availability, as often observed in soils. Nodulated N_2_-fixing legumes generally have a greater demand for mineral nutrients than their unnodulated, NO_3_-fed counterparts [45]. Earlier studies have, in fact, shown that high N_2_-fixing symbioses generally accumulated greater nutrient elements in shoots than their low-fixing counterparts [43], implying that rhizobia do have a say in the mineral nutrition of their host plants. In this study, rhizobial isolate TUTGmGH3 effectively nodulated and increased the levels of N, P and Cu in pigeonpea and mungbean in 2022. It is interesting to note that the results in the present study revealed a significant difference among all the parameters tested over the two cropping periods (Table S3 and S4).

## 4. Materials and Methods

### 4.1. Site Description

The experiment was conducted under glasshouse conditions at the Tshwane University of Technology, Pretoria campus, from October to December 2021 and January to April 2022.

### 4.2. Origin of the Legumes, Experimental Design and Planting

Bambara groundnut (*Vigna subterranea*), common bean (*Phaseolus vulgaris*), jack bean (*Canavalia ensiformis*), Winged bean (*Psophocarpus tetragonolobus*) and Velvet bean (*Mucuna pruriens*) seeds were obtained from Etsala National Seed Co., Ltd., Malkerns, Swaziland; cowpea (*Vigna unguiculata*), soybean (*Glycine max*), mungbean (*Vigna radiata*) and pigeonpea (*Cajanus caja*) seeds from the International Institute of Tropical Agriculture, Mozambique (IITA-Mozambique, Maputo, Mozambique); Kersting’s groundnut seeds were obtained from the University for Development Studies (UDS) in Tamale, Ghana. The commercial inoculant strains used for cowpea, Bambara groundnut and Kersting’s groundnut were *Bradyrhizobium* strain CB756, common bean *Rhizobium leguminosarum* strain UD5 and soybean *Bradyrhizobium japonicum* strain WB74.

**Figure 3:**
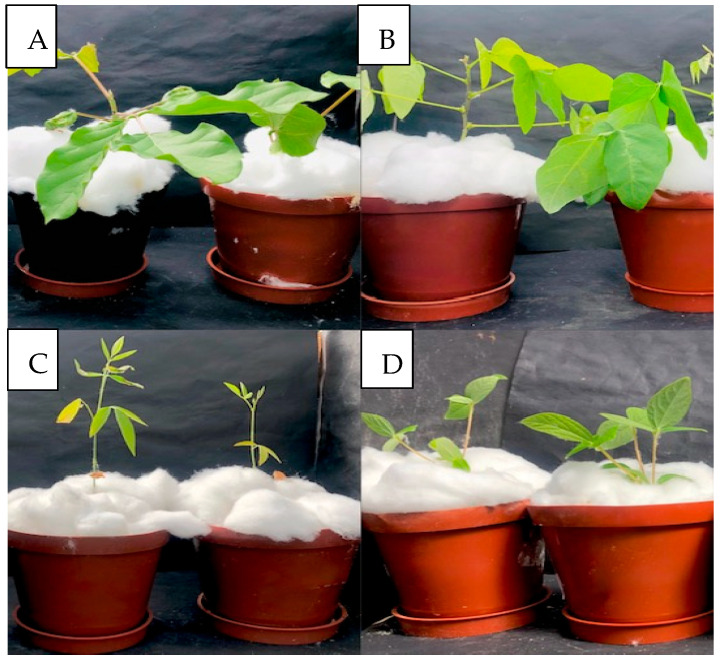

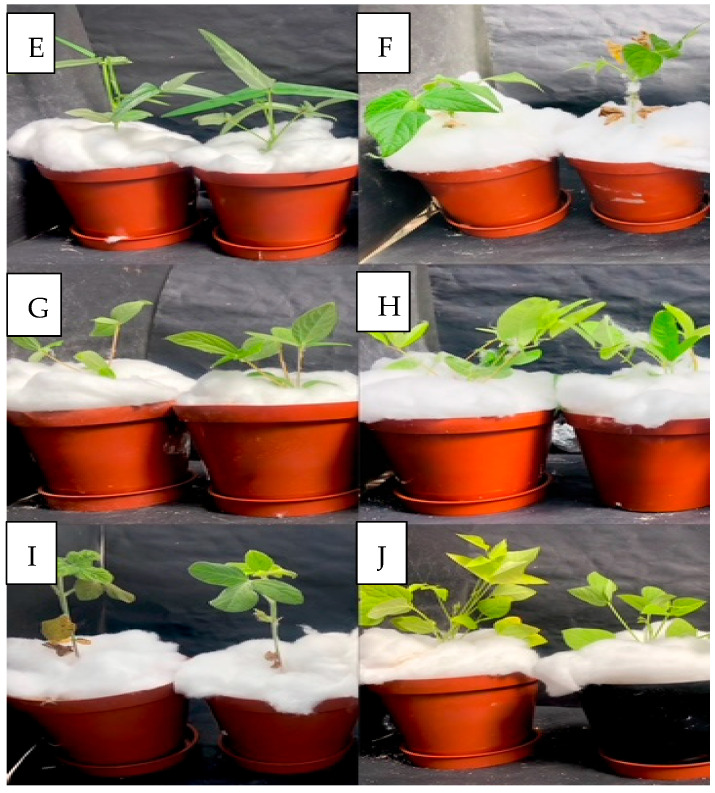
Images of inoculated legume species used in the study: (**A**) Jack bean, (**B**) Velvet bean, (**C**) pigeonpea, (**D**) mungbean, (**E**) cowpea, (**F**) common bean, (**G**) Kersting’s groundnut, (**H**) Bambara groundnut, (**I**) soybean and (**J**) Winged bean.

Prior to planting, seeds of 10 test legume species, two genotypes per crop (cowpea, Bambara groundnut, cowpea, Kersting’s groundnut, common bean, soybean, Winged bean, Velvet bean, pigeonpea, mungbean and Jack bean) were surface-sterilized by immersing them in 95% ethanol for 5 to 10 s, then in 3% sodium hypochlorite for 2–3 min, and rinsed six times with sterile distilled water [46]. The seeds were germinated in autoclaved sand contained in plastic pots (1200 cm^3^) covered with sterile non-absorbent cotton wool to avoid moisture loss and contamination. Three sterilized seeds were planted per pot and thinned out to one seedling per pot after germination. The legume seedlings were inoculated with 1 mL of broth culture (15 isolates) per isolate, grown to the exponential phase (106–107 cells/mL) using sterilized micro pipettes. The plants were irrigated with N-free nutrient solution (Table S5), and when necessary, with sterile water. Uninoculated 5 mM KNO3-fed and commercial Bradyrhizobium inoculant were included as controls. Three replicate pots were used per isolate. The pots were arranged in a randomized block design. The plants were harvested at 60 days after planting to assess root nodulation. Each plant was separated into shoots and nodulated roots. The shoots were oven dried at 65 °C for 72 h, weighed, ground into fine powder (0.50 mm sieve), and stored in plastic Ziploc bags prior to mineral analysis. Data on plant biomass components, such as shoot, root, and nodule biomass, as well as the number of nodules per plant, can be found at Msiza et al. [47]. Details of images or pictures of the different legume species used in the study are presented in (Figure 3A–J).

### 4.3. Bacteria Isolation from Root Nodules

The isolation of bacteria from root nodules of legume plants was performed as described by Somasegaran and Hoben [46]. Healthy nodules were selected, and the bacteria isolated. The nodule suspensions were streaked onto yeast–mannitol agar (YMA) plates and incubated at 28 °C. The plates were monitored daily to record the time taken for colonies to appear. In instances where we did not obtain single colonies, re-streaking was performed to obtain single colonies.

### 4.4. Photosynthetic Measurements in Plant Leaves

Leaf photosynthetic rates (A), stomatal conductance (gs), transpiration (E), and internal CO_2_ concentration (Ci) were measured using a portable infrared red gas analyzer, version 6.2 (LI 6400XT, Lincoln, NE, USA). Gas exchange measurements were performed using the following chamber conditions: photosynthetic flux density 1000 μmol m^−2^ s^−1^, reference CO_2_ concentration 400 μmol mol^−1^ and flow rate 500 μmol s^−1^. Intrinsic water-use efficiency (WUEi) was computed as the ratio of net photosynthetic rate (A) to stomatal conductance (gs) [48].

$$WUE=\frac{A}{gs}$$

### 4.5. Determination of Mineral Nutrients in the Shoots of Test Legume Species

To measure P, K, Ca, Mg, S, Fe, Zn, Mn, Cu and B in legume shoots, 1 g of ground plant sample was ashed overnight in a porcelain crucible at 500 °C. This was followed by dissolving the ash in 5 mL of analytical grade 6 M HCl and placing it in an oven at 50 °C for 30 min, after which 35 mL of de-ionized water was added. The mixture was filtered through Whatman No. 1 filter paper, and the mineral element concentrations in plant extracts were determined for each of the three replicate samples using inductively coupled plasma mass spectrometry (IRIS/AP HR DUO Thermo Electron Corporation, Franklin, MA, USA).

### 4.6. Statistical Analysis

All data collected were subjected to a normality test before being subjected to analysis of variance (ANOVA) using statistical software version 10.1 (StatSoft Inc., 2011, Tulsa, OK, USA). A 1-way ANOVA was used to compare photosynthetic parameters and mineral elements and to determine the mean differences. Duncan’s multiple range test at the *p* ≤ 0.05 level was used to separate means that showed a significant difference. All graphs and figures were constructed using Microsoft Excel.

## 5. Conclusions

Taken together, the results of the present study have demonstrated that rhizobial strains significantly influence the photosynthetic performance of their host plants, with variations largely dependent on strain-host compatibility and symbiotic efficiency. Isolate TUTGmGH2 notably enhanced the accumulation of P, K, Mg, Zn, Cu, and Mn in soybean and Winged bean, indicating a strong role in improving plant mineral nutrition. This highlights its potential and could be used for inoculant formulation to enhance crop productivity and support sustainable agriculture.

## Figures and Tables

**Table 1:** Photosynthetic rate (A), stomatal conductance (gs), transpiration rate (E) and WUE of native rhizobial isolates at Tshwane University of Technology under glasshouse conditions in 2021. Mean values with dissimilar letters in a column are significantly different at *p* < 0.05.

	Treatments	A	gs	E	WUE
		µmol CO_2_ m^−2^ s^−1^	mmol m^−1^·s^−1^	mmol m^−1^·H_2_O	mmol CO_2_ m^−1^·H_2_O
**Cowpea**	TUTVuSA1	17.46 ± 0.28 b	0.31 ± 0.05 d	0.69 ± 0.00 f	56.23 ± 11.80 g
cv. IT10K-817-3	TUTVuSA2	18.92 ± 0.03 a	0.38 ± 0.09 a	0.71 ± 0.01 e	49.78 ± 16.31 dh
	TUTVuSA3	15.76 ± 0.15 c	0.23 ± 0.03 ef	0.68 ± 0.01 g	68.52 ± 9.26 f
	TUTPvES3	17.85 ± 0.48 b	0.05 ± 0.00 h	0.23 ± 0.01 h	357.84 ± 18.76 a
	TUTGmGH1	19.07 ± 0.49 a	0.25 ± 0.02 e	0.68 ± 0.01 g	76.28 ± 4.95 e
	TUTGmGH3	17.45 ± 0.42 b	0.19 ± 0.01 g	0.69 ± 0.01 fg	91.84 ± 0.77 d
	COMMERCIAL INOC	13.80 ± 0.49 e	0.36 ± 0.00 b	1.31 ± 0.00 b	38.33 ± 1.80 j
	NITRATE	15.41 ± 0.12 cd	0.34 ± 0.07 c	1.55 ± 0.11 a	45.32 ± 8.24 i
	UNINOCULATED	14.75 ± 0.12 de	0.08 ± 0.00 h	1.16 ± 0.00 c	184.37 ± 5.66 c
	***F-statistics***	62.83 **	16.96 ***	118.73 **	100.13 **
**Bambara groundnut**	TUTVsES1	12.34 ± 0.40 de	0.14 ± 0.00 a	5.70 ± 0.09 a	88.14 ± 5.19 ef
LR.SSD5	TUTVsES2	10.92 ± 0.00 e	0.13 ± 0.00 b	3.67 ± 0.00 b	84.04 ± 0.00 f
	TUTVsES3	9.60 ± 0.00 f	0.08 ± 0.00 ef	2.54 ± 0.00 c	120.41 ± 0.00 e
	TUTGmGH1	19.00 ± 0.00 a	0.11 ± 0.00 c	0.60 ± 0.00 d	172.72 ± 2.79 c
	TUTGmGH2	14.03 ± 0.00 c	0.11 ± 0.00 c	0.50 ± 0.00 f	127.54 ± 4.09 de
	TUTGmGH3	10.11 ± 0.00 e	0.14 ± 0.00 a	0.54 ± 0.00 e	72.21 ± 0.00 i
	TUTMgSA3	10.14 ± 0.22 e	0.07 ± 0.00 f	3.58 ± 0.05 b	144.85 ± 4.66 d
	COMMERCIAL INOC	13.36 ± 0.00 d	0.06 ± 0.00 fg	0.57 ± 0.00 ef	222.66 ± 0.00 a
	NITRATE	15.79 ± 0.00 b	0.08 ± 0.01 ef	0.48 ± 0.00 g	197.37 ± 13.78 b
	UNINOCULATED	10.92 ± 0.00 e	0.13 ± 0.00 e	3.76 ± 0.67 b	84.04 ± 0.00 f
	***F-statistics***	220.83 **	242.38 **	157.77 **	90.34 **
**Kersting’s groundnut**	TUTMgSA1	9.35 ± 0.89 f	0.09 ± 0.01 i	2.64 ± 0.16 a	103.56 ± 6.21 f
LR.Puffeun	TUTMgSA2	16.80 ± 0.82 d	0.13 ± 0.01 dg	0.30 ± 0.08 g	129.23 ± 7.83 c
	TUTMgSA3	16.91 ± 0.31 d	0.27 ± 0.02 b	0.18 ± 0.01 h	62.62 ± 3.22 h
	TUTVuSA3	13.57 ± 0.89 e	0.31 ± 0.03 a	1.80 ± 0.08 b	43.77 ± 6.04 i
	TUTPvES1	17.58 ± 0.73 c	0.13 ± 0.00 dde	0.50 ± 0.01 e	135.23 ± 9.09 bc
	TUTPvES3	14.77 ± 1.02 de	0.12 ± 0.01 e	0.48 ± 0.01 f	123.08 ± 1.32 d
	TUTGmGH2	20.66 ± 0.05 b	0.15 ± 0.00 d	1.26 ± 0.00 d	137.78 ± 1.62 b
	COMMERCIAL INOC	22.09 ± 0.26 a	0.11 ± 0.00 f	1.71 ± 0.04 bc	200.81 ± 2.86 a
	NITRATE	16.16 ± 0.28 d	0.16 ± 0.00 c	1.53 ± 0.16 c	101.58 ± 2.43 g
	UNINOCULATED	17.01 ± 0.37 c	0.16 ± 0.00 c	1.71 ± 0.03 bc	106.31 ± 1.89 e
	***F-statistics***	32.25 ***	20.87 ***	43.14 **	29.68 ***
**Common bean**	TUTPvES1	7.85 ± 0.26 c	0.06 ± 0.00 b	1.69 ± 0.06 b	130.83 ± 11.88 e
cv. NUA 734	TUTPvES2	6.35 ± 0.00 d	0.04 ± 0.00 c	2.86 ± 0.07 a	158.75 ± 0.09 a
	TUTPvES3	7.96 ± 0.51 c	0.06 ± 0.00 b	1.81 ± 0.04 b	132.66 ± 4.64 d
	TUTGmGH2	8.62 ± 0.15 b	0.07 ± 0.00 a	1.97 ± 0.04 b	123.14 ± 3.56 f
	TUTGmGH3	8.99 ± 0.32 b	0.06 ± 0.00 b	2.11 ± 0.00 a	149.83 ± 2.16 b
	COMMERCIAL INOC	7.62 ± 0.00 c	0.07 ± 0.00 a	1.96 ± 0.00 b	108.85 ± 0.00 g
	NITRATE	9.41 ± 0.35 a	0.07 ± 0.00 a	2.99 ± 0.01 a	134.43 ± 12.41 c
	UNINOCULATED	7.44 ± 0.16 c	0.07 ± 0.00 a	2.51 ± 0.20 a	106.29 ± 6.66 h
	***F-statistics***	28.91 ***	6.87 ***	65.15 **	4.18 ***
**Soybean**	TUTGmGH1	17.65 ± 0.21 e	0.27 ± 0.00 a	0.67 ± 0.01 f	65.37 ± 0.93 f
cv. TGX1740-2F	TUTGmGH2	15.51 ± 0.26 f	0.12 ± 0.05 f	0.48 ± 0.01 h	129.25 ± 3.83 d
	TUTGmGH3	17.17 ± 0.40 e	0.15 ± 0.00 b	0.77 ± 0.01 c	114.46 ± 3.15 ef
	TUTVuSA1	15.05 ± 0.39 f	0.10 ± 0.01 hi	0.46 ± 0.01 i	150.06 ± 21.89 b
	TUTMgSA3	21.16 ± 1.16 b	0.13 ± 0.01 e	0.63 ± 0.02 g	162.76 ± 13.47 a
	TUTVsES2	25.52 ± 1.09 a	0.10 ± 0.00 g	0.80 ± 0.01 b	255.02 ± 9.60 ab
	TUTPvES2	19.32 ± 0.035 c	0.14 ± 0.00 c	0.75 ± 0.01 d	138.17 ± 1.26 c
	COMMERCIAL INOC	18.07 ± 0.01 d	0.12 ± 0.00 f	0.67 ± 0.01 f	150.58 ± 0.75 b
	NITRATE	17.65 ± 0.33 e	0.14 ± 0.00 c	0.67 ± 0.01 f	126.07 ± 2.75 e
	UNINOCULATED	13.87 ± 0.33 g	0.10 ± 0.00 g	0.99 ± 0.01 a	138.69 ± 3.24 c
	***F-statistics***	53.69 **	20.69 ***	164.18 **	1.71 ns
**Winged bean**	TUTGmGH1	13.00 ± 0.53 c	0.36 ± 0.01 b	1.09 ± 0.00 d	36.11 ± 2.06 f
cv.VRWB 4A	TUTGmGH2	11.24 ± 0.29 e	0.46 ± 0.02 a	1.10 ± 0.00 c	24.43 ± 0.23 g
	TUTGmGH3	12.13 ± 0.39 d	0.15 ± 0.00 e	1.48 ± 0.00 a	80.86 ± 2.33 d
	TUTMgSA3	17.21 ± 0.33 b	0.32 ± 0.00 c	0.61 ± 0.01 f	53.78 ± 1.03 e
	TUTVsES1	19.84 ± 0.03 a	0.23 ± 0.00 d	0.78 ± 0.03 e	86.26 ± 0.82 c
	TUTPvES3	19.10 ± 0.35 a	0.14 ± 0.01 f	1.28 ± 0.01 b	136.43 ± 8.93 a
	UNINOCULATED	12.11 ± 0.31 d	0.10 ± 0.00 g	0.61 ± 0.01 f	121.01 ± 2.11 b
	***F-statistics***	25.58 ***	253.26 **	326.71 **	47.15 ***
**Velvet bean**	TUTGmGH1	9.35 ± 0.19 b	0.05 ± 0.00 b	1.75 ± 0.00 e	187.01 ± 3.53 d
cv. IIHR PS 1	TUTGmGH2	7.46 ± 0.00 d	0.05 ± 0.00 b	1.75 ± 0.00 e	149.02 ± 0.00 e
	TUTGmGH3	7.54 ± 0.00 d	0.06 ± 0.00 a	1.81 ± 0.00 c	125.66 ± 0.00 ef
	TUTVuSA1	8.01 ± 0.27 c	0.04 ± 0.00 c	1.87 ± 0.01 b	200.25 ± 7.57 c
	TUTMgSA3	7.06 ± 0.14 d	0.06 ± 0.00 a	1.79 ± 0.06 d	117.67 ± 2.38 f
	TUTVsES1	4.09 ± 0.06 f	0.02 ± 0.00 d	1.81 ± 0.04 c	204.05 ± 10.21 b
	TUTPvES2	12.45 ± 0.35 a	0.05 ± 0.00 b	2.37 ± 0.03 a	249.00 ± 6.90 a
	TUTPvES3	5.99 ± 0.14 e	0.06 ± 0.00 a	2.87 ± 0.04 a	99.83 ± 0.96 g
	UNINOCULATED	5.99 ± 0.09 e	0.05 ± 0.00 b	1.75 ± 0.00 e	119.08 ± 2.38 f
	***F-statistics***	96.27 **	363.13 **	127.96 **	78.76 **
**Jack bean**	TUTVuSA2	5.81 ± 0.17 e	0.03 ± 0.00 d	2.83 ± 0.32 a	193.66 ± 5.11 d
cv. Accession 493	TUTMgSA3	14.56 ± 0.09 a	0.07 ± 0.00 a	2.15 ± 0.00 b	208.00 ± 0.55 cd
	TUTVsES1	8.79 ± 0.10 c	0.04 ± 0.00 cd	2.25 ± 0.02 ab	219.75 ± 1.19 c
	TUTVsES2	7.20 ± 0.16 d	0.06 ± 0.00 b	2.89 ± 0.04 a	120.00 ± 1.21 e
	TUTVsES3	9.49 ± 0.30 b	0.05 ± 0.00 c	2.01 ± 0.00 c	189.08 ± 6.51 d
	TUTPvES2	8.61 ± 0.4 c	0.03 ± 0.00 d	2.32 ± 0.01 ab	287.01 ± 9.01 a
	TUTPvES3	5.28 ± 0.14 e	0.02 ± 0.00 e	2.83 ± 0.02 a	264.00 ± 5.54 b
	UNINOCULATED	5.81 ± 0.11 e	0.03 ± 0.00 d	1.77 ± 0.01 d	193.67 ± 1.01 d
	***F-statistics***	476.57 **	96.27 **	518.1 **	138.43 **
**Pigeonpea**	TUTVuSA1	14.42 ± 0.06 b	0.11 ± 0.00 e	0.40 ± 0.02 d	131.09 ± 2.44 c
ICEAP500557	TUTVuSA2	17.27 ± 0.24 a	0.12 ± 0.00 d	0.39 ± 0.00 e	143.92 ± 1.41 b
	TUTMgSA1	13.89 ± 0.97 c	0.09 ± 0.00 g	0.43 ± 0.03 c	154.33 ± 0.09 a
	TUTPvES2	17.34 ± 0.03 a	0.24 ± 0.02 b	0.08 ± 0.00 h	72.25 ± 4.81 g
	TUTGmGH1	7.75 ± 0.37 e	0.10 ± 0.00 f	0.37 ± 0.03 f	77.05 ± 0.55 f
	TUTGmGH2	17.13 ± 0.01 a	0.27 ± 0.00 a	0.91 ± 0.02 a	63.44 ± 0.50 h
	TUTGmGH3	13.93 ± 0.56 c	0.14 ± 0.00 c	0.55 ± 0.02 b	99.05 ± 1.30 d
	UNINOCULATED	8.77 ± 0.31 d	0.09 ± 0.00 g	0.04 ± 0.00 g	97.44 ± 2.63 e
	***F-statistics***	103.53 **	470.27 **	123.98 **	720.27 **
**Mungbean**	TUTVuSA2	13.97 ± 0.00 c	0.13 ± 0.00 h	1.35 ± 0.00 c	107.46 ± 2.11 a
cv. VC1973A	TUTMgSA2	15.97 ± 0.48 b	0.18 ± 0.00 d	0.49 ± 0.02 e	88.72 ± 1.63 bc
	TUTMgSA3	12.59 ± 0.70 d	0.33 ± 0.00 a	1.85 ± 0.08 a	38.15 ± 0.64 f
	TUTPvES1	9.89 ± 0.48 e	0.12 ± 0.00 f	1.60 ± 0.08 b	82.42 ± 3.68 c
	TUTGmGH1	15.45 ± 0.06 b	0.17 ± 0.01 e	0.47 ± 0.06 f	90.88 ± 3.47 b
	TUTGmGH3	13.36 ± 0.11 c	0.25 ± 0.00 b	0.95 ± 0.00 d	53.44 ± 0.31 e
	UNINOCULATED	16.27 ± 0.00 a	0.24 ± 0.00 c	0.26 ± 0.00 g	67.79 ± 1.11 d
	***F-statistics***	61.37 **	162.19 **	229.23 **	79.75 **

Values (Mean ± SE) with dissimilar letters in a column are significant at ** *p* ≤ 0.01, *** *p* ≤ 0.001 and ns = not significant.

**Table 2:** Photosynthetic rate (A), stomatal conductance (gs), transpiration rate (E) and WUE of native rhizobial isolates at Tshwane University of Technology under glasshouse conditions in 2022. Mean values with dissimilar letters in a column are significantly different at *p* < 0.05.

	Treatments	A	gs	E	WUE
		µmol CO_2_ m^−2^ s^−1^	mmol m^−1^·s^−1^	mmol m^−2^·H_2_O	mmol CO_2_ m^−1^·H_2_O
**Cowpea**	TUTVuSA1	6.93 ± 0.90 f	0.10 ± 0.00 d	0.11 ± 0.03 h	69.03 ± 10.12 hi
cv. IT10K-866-1	TUTVuSA2	7.39 ± 1.22 e	0.06 ± 0.01 g	0.86 ± 0.16 b	123.17 ± 6.97 ef
	TUTVuSA3	10.74 ± 1.42 c	0.13 ± 0.03 b	2.26 ± 0.86 a	82.62 ± 16.47 h
	TUTVsES1	7.94 ± 0.32 e	0.11 ± 0.01 c	0.50 ± 0.02 d	89.55 ± 5.57 g
	TUTPvES3	13.14 ± 0.69 a	0.08 ± 0.01 f	0.86 ± 0.31 b	164.25 ± 2.48 d
	TUTGmGH1	5.47 ± 0.16 g	0.02 ± 0.00 i	0.20 ± 0.00 c	273.05 ± 8.64 b
	TUTGmGH2	8.39 ± 0.62 d	0.03 ± 0.00 h	0.74 ± 0.02 c	279.67 ± 16.21 a
	TUTGmGH3	8.67 ± 0.25 d	0.06 ± 0.00 g	0.48 ± 0.04 e	144.05 ± 10.09 e
	COMMERCIAL INOC	11.06 ± 0.40 b	0.09 ± 0.002 e	0.40 ± 0.00 g	122.88 ± 2.53 f
	NITRATE	10.86 ± 0.99 c	0.06 ± 0.00 g–j	0.75 ± 0.01 bc	181.00 ± 4.72 c
	UNINOCULATED	8.89 ± 0.22 d	0.18 ± 0.00 a	0.44 ± 0.04 f	49.39 ± 2.20 i
	***F-statistics***	10.93 ***	22.88 ***	3.80 ***	61.99 **
**Bambara groundnut**	TUTVsES1	6.12 ± 0.11 g	0.12 ± 0.00 b	0.35 ± 0.02 bc	51.00 ± 0.49 j
LR. SSD8	TUTVsES2	11.06 ± 0.40 b	0.15 ± 0.01 a	0.40 ± 0.00 b	73.73 ± 1.96 i
	TUTVsES3	9.16 ± 0.74 d	0.09 ± 0.00 d	0.48 ± 0.04 ab	101.77 ± 7.02 g
	TUTMgSA3	8.72 ± 0.64 e	0.10 ± 0.00 c	0.49 ± 0.05 a	87.02 ± 4.49 h
	TUTPvES2	5.52 ± 0.59 h	0.05 ± 0.01 h	0.11 ± 0.02 f	110.04 ± 4.97 ef
	TUTGmGH1	10.21 ± 0.46 c	0.08 ± 0.00 e	0.38 ± 0.00 c	127.63 ± 8.48 d
	TUTGmGH2	6.87 ± 0.05 g	0.05 ± 0.00 h	0.11 ± 0.01 e	137.04 ± 4.76 a
	TUTGmGH3	10.53 ± 0.73 c	0.08 ± 0.00 e	0.19 ± 0.01 d	131.63 ± 4.20 b
	COMMERCIAL INOC	13.03 ± 1.52 a	0.10 ± 0.01 c	0.40 ± 0.01 b	130.03 ± 1.77 bc
	NITRATE	7.72 ± 0.50 f	0.06 ± 0.00 g	0.18 ± 0.02 de	128.67 ± 7.23 c
	UNINOCULATED	9.01 ± 0.08 d	0.08 ± 0.00 e	0.48 ± 0.04 ab	112.63 ± 6.25 e
	***F-statistics***	6.19 ***	53.72 **	7.71 ***	49.92 **
**Kersting’s groundnut**	TUTMgSA1	6.88 ± 0.07 i	0.14 ± 0.01 a	0.33 ± 0.01 f	49.14 ± 2.01 i
LR. Dowie	TUTMgSA2	9.53 ± 0.24 f	0.06 ± 0.00 f	0.53 ± 0.00 d	158.83 ± 8.28 d
	TUTMgSA3	14.86 ± 0.93 b	0.14 ± 0.01 a	0.36 ± 0.01 e	106.14 ± 2.82 g
	TUTVuSA3	7.31 ± 0.09 h	0.12 ± 0.00 b	0.33 ± 0.01 f	60.92 ± 0.16 h
	TUTPvES3	10.39 ± 0.09 de	0.05 ± 0.00 g	1.30 ± 0.00 a	207.08 ± 1.73 a
	TUTGmGH2	16.45 ± 0.10 a	0.10 ± 0.00 d	0.88 ± 0.30 b	164.05 ± 1.94 de
	TUTGmGH3	12.05 ± 0.33 c	0.06 ± 0.00 f	0.20 ± 0.01 h	200.83 ± 5.70 c
	COMMERCIAL INOC	8.11 ± 0.14 g	0.04 ± 0.00 h	0.14 ± 0.01 i	202.75 ± 2.93 b
	NITRATE	8.81 ± 0.91 g	0.07 ± 0.00 e	0.64 ± 0.01 c	125.86 ± 11.71 e
	UNINOCULATED	11.96 ± 0.44 d	0.11 ± 0.00 c	0.26 ± 0.03 g	108.73 ± 3.26 f
	***F-statistics***	60.23 **	109.39 **	24.37 ***	93.58 **
**Common bean**	TUTPvES1	17.56 ± 0.32 a	0.12 ± 0.00 b	0.55 ± 0.00 d	146.33 ± 2.66 d
cv. NUA 721	TUTPvES2	15.21 ± 0.25 b	0.17 ± 0.01 a	0.76 ± 0.02 a	89.47 ± 5.18 g
	TUTPvES3	12.87 ± 0.44 c	0.08 ± 0.00 e	0.46 ± 0.01 e	158.78 ± 5.46 c
	TUTGmGH1	8.44 ± 0.32 e	0.03 ± 0.00 g	0.63 ± 0.02 b	266.96 ± 10.46 a
	TUTGmGH3	6.52 ± 0.57 f	0.10 ± 0.00 c	0.11 ± 0.01 i	68.20 ± 4.41 h
	COMMERCIAL INOC	12.74 ± 0.58 c	0.06 ± 0.00 f	0.34 ± 0.02 h	227.11 ± 5.71 b
	NITRATE	9.38 ± 0.34 d	0.10 ± 0.00 c	0.36 ± 0.02 f	91.96 ± 3.11 f
	UNINOCULATED	9.60 ± 0.86 d	0.09 ± 0.00 d	0.60 ± 0.08 c	107.24 ± 12.48 e
	***F-statistics***	53.15 **	178.91 **	25.89 ***	49.19 **
**Soybean**	TUTGmGH1	15.73 ± 0.53 a	0.07 ± 0.00 f	0.40 ± 0.00 f	241.07 ± 5.64 b
cv. TGX1937-1F	TUTGmGH2	9.89 ± 0.43 ef	0.10 ± 0.00 d	0.91 ± 0.00 b	100.88 ± 3.94 f
	TUTGmGH3	13.94 ± 0.45 b	0.04 ± 0.00 i	0.33 ± 0.01 g	313.41 ± 11.02 a
	TUTVuSA1	5.73 ± 0.46 h	0.11 ± 0.00 c	0.03 ± 0.00 i	50.25 ± 3.85 h
	TUTMgSA3	8.73 ± 0.07 f	0.06 ± 0.00 g	0.61 ± 0.00 d	141.68 ± 1.33 d
	TUTVsES2	11.32 ± 0.08 d	0.05 ± 0.00 h	0.63 ± 0.01 d	213.37 ± 5.15 c
	TUTPvES2	13.06 ± 0.79 bc	0.06 ± 0.00 g	0.36 ± 0.01 h	226.64 ± 10.76 bc
	TUTPvES3	8.34 ± 0.42 f	0.21 ± 0.00 a	0.74 ± 0.02 c	40.03 ± 2.07 i
	COMMERCIAL INOC	12.56 ± 0.17 c	0.12 ± 0.00 b	0.43 ± 0.02 e	108.60 ± 0.95 e
	NITRATE	10.07 ± 0.43 e	0.09 ± 0.00 e	1.16 ± 0.02 a	108.35 ± 5.77 e
	UNINOCULATED	7.18 ± 0.39 g	0.07 ± 0.00 f	0.26 ± 0.00 h	97.62 ± 8.08 g
	***F-statistics***	66.35 **	510.66 **	879.32 **	110.12 **
**Velvet bean**	TUTVuSA1	7.69 ± 0.21 d	0.09 ± 0.00 d	0.23 ± 0.00 h	88.43 ± 3.91 f
cv. IIHR PS 2	TUTVuSA2	10.88 ± 0.21 c	0.10 ± 0.00 c	0.68 ± 0.00 d	107.22 ± 2.09 c
	TUTVuSA3	8.19 ± 0.09 e	0.16 ± 0.01 a	0.49 ± 0.00 d-f	51.04 ± 1.93 gh
	TUTMgSA1	5.61 ± 0.14 ij	0.04 ± 0.00 i	0.10 ± 0.00 h	132.52 ± 5.26 de
	TUTMgSA2	6.87 ± 0.05 f-h	0.07 ± 0.00 fg	0.18 ± 0.01 gh	104.84 ± 2.04 ef
	TUTMgSA3	11.53 ± 0.19 bc	0.06 ± 0.00 gh	0.36 ± 0.02 e-g	192.20 ± 8.53 ab
	TUTVsES1	7.78 ± 0.47 d	0.10 ± 0.00 c	0.63 ± 0.06 e	76.43 ± 4.58 g
	TUTVsES2	14.02 ± 0.47 a	0.09 ± 0.00 d	0.23 ± 0.02 gh	151.93 ± 0.65 cd
	TUTPvES2	11.62 ± 0.99 b	0.16 ± 0.00 a	3.04 ± 0.10 a	73.64 ± 8.38 h
	TUTPvES3	12.38 ± 0.18 a	0.13 ± 0.00 b	0.28 ± 0.01 g	95.95 ± 4.70 d
	TUTGmGH3	10.83 ± 0.35 c	0.05 ± 0.00 f	1.04 ± 0.01 c	222.45 ± 8.48 a
	NITRATE	6.55 ± 0.09 e	0.07 ± 0.00 e	1.87 ± 0.03 b	91.39 ± 2.30 e
	UNINOCULATED	4.79 ± 0.60 f	0.03 ± 0.00 g	0.51 ± 0.01 f	194.62 ± 46.34 b
	***F-statistics***	62.74 **	193.71 **	92.95 **	19.54 ***
**Jack bean**	TUTVuSA2	3.55 ± 0.14 e	0.05 ± 0.00 d	1.18 ± 0.01 a	72.60 ± 6.18 e
cv. Accession 498	TUTVsES2	11.89 ± 0.64 b	0.10 ± 0.00 a	0.55 ± 0.00 d	113.95 ± 4.21 c
	TUTGmGH3	8.73 ± 0.7 d	0.09 ± 0.00 b	0.63 ± 0.02 c	95.29 ± 0.86 d
	NITRATE	12.38 ± 0.18 a	0.10 ± 0.00 a	0.27 ± 0.00 e	122.07 ± 1.75 b
	UNINOCULATED	10.53 ± 0.16 c	0.06 ± 0.00 c	0.76 ± 0.01 b	164.36 ± 2.20 a
	***F-statistics***	157.03 **	293.66 **	77.41 **	174.72 **
**Pigeonpea**	TUTVuSA1	14.22 ± 0.36 c	0.17 ± 0.01 a	0.96 ± 0.00 c	82.61 ± 2.43 h
cv. ICEAP00850	TUTMgSA1	11.59 ± 0.31 d	0.11 ± 0.00 b	0.64 ± 0.03 f	107.83 ± 3.03 f
	TUTVsES1	7.94 ± 0.58 g	0.05 ± 0.00 f	0.83 ± 0.01 e	165.78 ± 3.88 c
	TUTPvES2	14.20 ± 0.18 c	0.08 ± 0.01 e	1.55 ± 0.03 b	185.87 ± 16.99 a
	TUTGmGH2	9.48 ± 0.20 f	0.11 ± 0.00 b	1.84 ± 0.6 a	88.62 ± 1.96 g
	TUTGmGH3	7.27 ± 0.16 g	0.08 ± 0.01 e	0.86 ± 0.03 d	88.79 ± 5.94 g
	NITRATE	15.36 ± 0.07 b	0.11 ± 0.00 b	0.96 ± 0.01 c	136.35 ± 0.53 d
	UNINOCULATED	10.32 ± 0.25 e	0.09 ± 0.00 d	0.52 ± 0.03 g	115.08 ± 6.61 e
	***F-statistics***	137.95 **	147.37 **	179.60 **	64.02 **
**Mungbean**	TUTMgSA2	12.59 ± 0.03 b	0.10 ± 0.00 a	0.33 ± 0.03 e	123.97 ± 0.30 d
cv. VC6 153 (B-20P	TUTMgSA3	15.06 ± 1.02 a	0.09 ± 0.00 b	0.47 ± 0.01 c	163.03 ± 8.08 b
	TUTPvES1	9.92 ± 0.19 bc	0.10 ± 0.00 a	1.67 ± 0.06 a	99.80 ± 3.96 f
	TUTGmGH2	5.60 ± 0.28 e	0.03 ± 0.00 d	0.09 ± 0.00 f	201.26 ± 7.92 a
	TUTGmGH3	9.88 ± 0.04 c	0.09 ± 0.00 b	0.44 ± 0.01 cd	111.50 ± 7.05 e
	NITRATE	12.84 ± 0.75 b	0.08 ± 0.00 c	0.76 ± 0.02 b	151.20 ± 5.37 c
	UNINOCULATED	8.44 ± 0.11 d	0.10 ± 0.00 a	0.34 ± 0.02 d	83.48 ± 1.11 g
	***F-statistics***	47.54 **	33.82 ***	204.77 **	61.58 **

Values (Mean ± SE) with dissimilar letters in a column are significant at ** *p* ≤ 0.01, *** *p* ≤ 0.001 and ns = not significant.

## Data Availability

The original contributions presented in this study are included in the article/Supplementary Materials. Further inquiries can be directed to the corresponding author.

## Supplementary Materials

The following supporting information can be downloaded at: https://www.mdpi.com/article/10.3390/plants15101478/s1, Table S1: Photosynthetic rate (A), stomatal conductance (gs), transpiration rate (E) and WUE of native rhizobial isolates at Tshwane University of Technology under glasshouse conditions in 2021. Mean values with dissimilar letters in a column are significantly different at *p* < 0.05. Table S2: Photosynthetic rate (A), stomatal conductance (gs), transpiration rate (E) and WUE of native rhizobial isolates at Tshwane University of Technology under glasshouse conditions in 2022. Mean values with dissimilar letters in a column are significantly different at *p* < 0.05. Table S3: accumulation of nutrient elements in the shoots of diverse legume species nodulated by native rhizobial isolates in the glasshouse in 2021. Table S4: accumulation of nutrient elements in the shoots of diverse legume species nodulated by native rhizobial isolates in the glasshouse in 2022. Table S5: N-free plant nutrient solution used in this study.

## Abbreviations

The following abbreviations are used in this manuscript:

GS	Stomatal Conductance
WUE	Water-Use Efficiency
E	Transpiration
