# Supplementary material for: Water-Use Efficiency and Mineral Nutrition of Diverse Legume Species Nodulated by Different Native Rhizobial Isolates: Do Rhizobia Have a Say in the Mineral Nutrition of Their Host Plants?
Source: Plants (Basel). 2026 May 12;15(10):1478. doi: 10.3390/plants15101478 (PMC13210493; doi:10.3390/plants15101478)
Supplement: Supplementary file 1 [file plants-15-01478-s001.zip › plants-4266311-supplementary.pdf]

Table S1: Photosynthetic rate (A), stomatal conductance (gs), transpiration rate (E) and WUE of native rhizobial isolates at Tshwane University of Technology under glasshouse conditions in 2021. Mean values with dissimilar letters in a column are significantly different at p<0.05.

| | **Isolate numbers** | **A** | **gs** | **E** | **WUE** |
|---|---|---|---|---|---|
| | | **μmol $CO_2$ $m^{-2}s^{-1}$** | **mmol $m^{-1}.s^{-1}$** | **mmol $m^{-1}.H_2O$** | **mmol CO2 $m^{-1}.H_2O$** |
| **Cowpea** | TUTVuSA1 | 17.46±0.28b | 0.31±0.05cf | 0.69±0.00h | 56.23±11.80cf |
| cv. IT10K-817-3 | TUTVuSA2 | 18.92±0.03a | 0.38±0.09bd | 0.71±0.01h | 49.78±16.31df |
| | TUTVuSA3 | 15.76±0.15c | 0.23±0.03fg | 0.68±0.01h | 68.52±9.26ce |
| | TUTMgSA1 | 12.88±0.28g | 0.42±0.03ac | 1.49±0.06bc | 30.66±3.49f |
| | TUTMgSA2 | 14.35±0.48ef | 0.36±0.01be | 1.42±0.02cd | 39.86±0.83ef |
| | TUTMgSA3 | 12.86±0.21g | 0.42±0.04ac | 1.34±0.02de | 30.61±3.93f |
| | TUTVsES1 | 10.98±0.11h | 0.28±0.04dg | 0.83±0.06g | 39.21±4.95ef |
| | TUTVsES2 | 13.73±0.08fg | 0.46±0.02ab | 1.62±0.09a | 30.17±1.05f |
| | TUTVsES3 | 13.63±0.16fg | 0.35±0.01be | 1.27±0.01ef | 38.94±0.38ef |
| | TUTPvES1 | 12.94±0.39g | 0.52±0.05a | 1.51±0.03ac | 24.88±3.26f |
| | TUTPvES2 | 18.97±0.35a | 0.06±0.01h | 0.25±0.02i | 316.16±30.92a |
| | TUTPvES3 | 17.85±0.48b | 0.05±0.00h | 0.23±0.01i | 357.84±18.76a |
| | TUTGmGH1 | 19.07±0.49a | 0.25±0.02eg | 0.68±0.01h | 76.28±4.95cd |
| | TUTGmGH2 | 15.77±0.11c | 0.08±0.00h | 0.86±0.01g | 197.13±8.65b |
| | TUTGmGH3 | 17.45±0.42b | 0.19±0.01g | 0.69±0.01h | 91.84±0.77c |
| | COMMERCIAL INOC | 13.80±0.49fg | 0.36±0.00be | 1.31±0.00de | 38.33±1.80ef |
| | NITRATE | 15.41±0.12cd | 0.34±0.07cf | 1.55±0.11ab | 45.32±8.24df |
| | UNINOCULATED | 14.75±0.12de | 0.08±0.00h | 1.16±0.00f | 184.37±5.66b |
| | ***F-statistics*** | 62.83** | 16.96*** | 118.73** | 100.13** |
| **Bambara** | TUTVuSA1 | 7.36±0.20o | 0.06±0.00f | 2.07±0.01e | 122.66±1.19d |
| **Groundnut** | TUTVuSA2 | 8.38±0.23jk | 0.07±0.00f | 3.57±0.25d | 119.54±4.63df |
| LR.SSD5 | TUTVuSA3 | 8.86±0.00ij | 0.07±0.00e | 2.28±0.00e | 126.57±0.00d |
| | TUTMgSA1 | 9.09±0.36hi | 0.09±0.00d | 4.11±0.21d | 101.76±6.14f |
| | TUTMgSA2 | 9.75±0.00g | 0.08±0.00d | 2.51±0.00e | 121.05±0.00d |
| | TUTMgSA3 | 10.14±0.22g | 0.07±0.00f | 3.58±0.05d | 144.85±4.66c |

| | | | | | |
|---|---|---|---|---|---|
| | TUTVsES1 | 12.34±0.40e | 0.14±0.00b | 5.70±0.09c | 88.14±5.19g |
| | TUTVsES2 | 10.92±0.00f | 0.13±0.00b | 3.67±0.00d | 84.04±0.00hi |
| | TUTVsES3 | 9.60±0.00gh | 0.08±0.00d | 2.54±0.00e | 120.41±0.00df |
| | TUTPvES1 | 13.87±0.35cd | 0.16±0.00a | 8.56±0.05a | 86.68±3.40hi |
| | TUTPvES2 | 8.13±0.44k | 0.07±0.00e | 6.34±0.00b | 116.15±5.85ef |
| | TUTPvES3 | 9.14±0.00hi | 0.04±0.00g | 2.34±0.00e | 228.05±0.00a |
| | TUTGmGH1 | 19.00±0.00a | 0.11±0.00c | 0.60±0.00f | 172.72±2.79b |
| | TUTGmGH2 | 14.03±0.00c | 0.11±0.00c | 0.50±0.00f | 127.54±4.09de |
| | TUTGmGH3 | 10.11±0.00g | 0.14±0.00b | 0.54±0.00f | 72.21±0.00i |
| | COMMERCIAL INOC | 13.36±0.00d | 0.06±0.00f | 0.57±0.00f | 222.66±0.00a |
| | NITRATE | 15.79±0.00b | 0.08±0.01d | 0.48±0.00f | 197.37±13.78a |
| | UNINOCULATED | 10.92±0.00f | 0.13±0.00b | 3.76±0.67d | 84.04±0.00hi |
| | ***F-statistics*** | 220.83** | 242.38** | 157.77** | 90.34** |
| **Kersting's groundnut** | TUTVuSA1 | 13.34±1.15g | 0.17±0.02d | 1.14±0.02d | 78.47±4.15fg |
| LR.Puffeun | TUTVuSA2 | 10.58±0.67h | 0.09±0.00hi | 1.52±0.31bc | 117.55±11.12cd |
| | TUTVuSA3 | 13.57±0.89g | 0.31±0.03a | 1.80±0.08b | 43.77±6.04i |
| | TUTMgSA1 | 9.35±0.89h | 0.09±0.01i | 2.64±0.16a | 103.56±6.21de |
| | TUTMgSA2 | 16.80±0.82cf | 0.13±0.01dg | 0.30±0.08gh | 129.23±7.83cd |
| | TUTMgSA3 | 16.91±0.31ce | 0.27±0.02b | 0.18±0.01h | 62.62±3.22hi |
| | TUTVsES1 | 20.05±0.20ab | 0.23±0.00c | 0.79±0.03e | 87.17±0.88eg |
| | TUTVsES2 | 17.99±0.24cd | 0.16±0.03de | 0.72±0.04ef | 112.43±21.01cd |
| | TUTVsES3 | 18.68±0.54bc | 0.12±0.00fi | 0.40±0.05f-h | 155.66±1.22b |
| | TUTPvES1 | 17.58±0.73cd | 0.13±0.00dh | 0.50±0.01e-h | 135.23±9.09c |
| | TUTPvES2 | 15.36±0.33eg | 0.22±0.00c | 0.58±0.01e-g | 69.81±2.69g |
| | TUTPvES3 | 14.77±1.02fg | 0.12±0.01ei | 0.48±0.01e-h | 123.08±1.32cd |
| | TUTGmGH1 | 22.06±0.90a | 0.17±0.01de | 1.38±0.09cd | 129.76±16.41c |
| | TUTGmGH2 | 20.66±0.05a | 0.15±0.00dg | 1.26±0.00cd | 137.78±1.62bc |
| | TUTGmGH3 | 21.99±0.69a | 0.11±0.00gi | 1.51±0.08bc | 199.91.04±6.57a |
| | COMMERCIAL INOC | 22.09±0.26a | 0.11±0.00gi | 1.71±0.04b | 200.81±2.86a |
| | NITRATE | 16.16±0.28ddf | 0.16±0.00df | 1.53±0.16bc | 101.58±2.43df |
| | UNINOCULATED | 17.01±0.37ce | 0.16±0.00de | 1.71±0.03b | 106.31±1.89df |
| | ***F-statistics*** | 32.25*** | 20.87*** | 43.14** | 29.68*** |

| | | | | | |
|---|---|---|---|---|---|
| **Common bean** cv. NUA 734 | TUTVuSA1 | 9.63±0.33c | 0.08±0.00ab | 2.27±0.14d | 120.38±1.37c-f |
| | TUTVuSA2 | 7.14±0.51ef | 0.05±0.00gh | 1.87±0.06fg | 142.08±13.99ab |
| | TUTVuSA3 | 7.55±0.15e | 0.06±0.00eg | 1.81±0.08fg | 125.83±7.25b-d |
| | TUTMgSA1 | 6.08±0.58gh | 0.07±0.01be | 1.83±0.07fg | 86.86±22.57f |
| | TUTMgSA2 | 7.39±0.33e | 0.07±0.01cf | 1.82±0.07fg | 105.57±9.06c-f |
| | TUTMgSA3 | 12.24±0.29a | 0.09±0.01a | 2.91±0.05b | 136.50±11.86bc |
| | TUTVsES1 | 9.39±0.20c | 0.08±0.01ac | 3.89±0.03a | 117.38±11.08c-f |
| | TUTVsES2 | 7.23±0.23ef | 0.06±0.00dg | 1.76±0.01fg | 120.05±10.60c-f |
| | TUTVsES3 | 10.78±0.38b | 0.08±0.00ad | 1.93±0.02e-g | 134.75±6.28bc |
| | TUTPvES1 | 7.85±0.26de | 0.06±0.00df | 1.69±0.06g | 130.83±11.88b-f |
| | TUTPvES2 | 6.35±0.00fg | 0.04±0.00h | 2.86±0.07b | 158.75±0.09a |
| | TUTPvES3 | 7.96±0.51de | 0.06±0.00dg | 1.81±0.04fg | 132.66±4.64b-e |
| | TUTGmGH1 | 5.18±0.26h | 0.05±0.00fg | 2.55±0.00c | 103.06±7.17ef |
| | TUTGmGH2 | 8.62±0.15cd | 0.07±0.00be | 1.97±0.04ef | 123.14±3.56b-e |
| | TUTGmGH3 | 8.99±0.32c | 0.06±0.00df | 2.11±0.00de | 149.83±2.16bc |
| | COMMERCIAL INOC | 7.62±0.00e | 0.07±0.00be | 1.96±0.00ef | 108.85±0.00d-f |
| | NITRATE | 9.41±0.35c | 0.07±0.00be | 2.99±0.01b | 134.43±12.41b-d |
| | UNINOCULATED | 7.44±0.16e | 0.07±0.00cf | 2.51±0.20c | 106.29±6.66c-f |
| | ***F-statistics*** | 28.91*** | 6.87*** | 65.15** | 4.18*** |
| **Soybean** cv. TGX1740-2F | TUTVuSA1 | 15.05±0.39gh | 0.10±0.01hi | 0.46±0.01j | 150.06±21.89ac |
| | TUTVuSA2 | 10.68±0.10i | 0.03±0.01j | 0.57±0.02h | 356.00±13.79ab |
| | TUTVuSA3 | 11.21±0.27i | 0.07±0.01ij | 0.46±0.01j | 160.14±17.12ac |
| | TUTMgSA1 | 19.04±0.27c-e | 0.11±0.00eh | 0.51±0.01i | 173.09±4.24ac |
| | TUTMgSA2 | 17.36±0.39f | 0.15±0.01cd | 0.72±0.01e | 115.73±4.65c |
| | TUTMgSA3 | 21.16±1.16b | 0.13±0.01ch | 0.63±0.02g | 162.76±13.47ac |
| | TUTVsES1 | 17.94±0.33d-f | 0.12±0.00dh | 0.63±0.02g | 145.75±4.47ac |
| | TUTVsES2 | 25.52±1.09a | 0.10±0.00gi | 0.80±0.01c | 255.02±9.60a |
| | TUTVsES3 | 19.13±0.39c-e | 0.23±0.00b | 0.90±0.01b | 83.17±2.19c |
| | TUTPvES1 | 20.03±0.49bc | 0.17±0.01c | 0.73±0.00e | 117.82±5.88c |
| | TUTPvES2 | 19.32±0.035cd | 0.14±0.00cf | 0.75±0.01de | 138.17±1.26bc |
| | TUTPvES3 | 17.33±0.03f | 0.22±0.00b | 0.58±0.01h | 78.77±1.13c |

| | | | | | |
|---|---|---|---|---|---|
| | TUTGmGH1 | 17.65±0.21ef | 0.27±0.00a | 0.67±0.01f | 65.37±0.93c |
| | TUTGmGH2 | 15.51±0.26g | 0.12±0.05dh | 0.48±0.01j | 129.25±3.83ac |
| | TUTGmGH3 | 17.17±0.40f | 0.15±0.00ce | 0.77±0.01cd | 114.46±3.15c |
| | COMMERCIAL INOC | 18.07±0.01d-f | 0.12±0.00dh | 0.67±0.01f | 150.58±0.75ac |
| | NITRATE | 17.65±0.33ef | 0.14±0.00cg | 0.67±0.01f | 126.07±2.75c |
| | UNINOCULATED | 13.87±0.33h | 0.10±0.00fi | 0.99±0.01a | 138.69±3.24bc |
| | ***F-statistics*** | 53.69** | 20.69*** | 164.18** | 1.71ns |
| **Winged bean** | TUTVuSA1 | 12.07±0.32f | 0.08±0.01j | 0.90±0.01h | 149.06±12.22a |
| cv.VRWB 4A | TUTVuSA2 | 13.61±0.79f | 0.10±0.00i | 0.97±0.01e | 136.01±7.74ab |
| | TUTVuSA3 | 17.45±0.28ce | 0.17±0.01f | 0.80±0.01hi | 102.64±7.62c |
| | TUTMgSA1 | 18.60±0.36bd | 0.13±0.00h | 0.78±0.00i | 143.08±3.48ab |
| | TUTMgSA2 | 15.87±0.55e | 0.22±0.00e | 0.61±0.02j | 72.14±2.67de |
| | TUTMgSA3 | 17.21±0.33de | 0.32±0.00c | 0.61±0.01j | 53.78±1.03f |
| | TUTVsES1 | 19.84±0.03ab | 0.23±0.00e | 0.78±0.03i | 86.26±0.82cd |
| | TUTVsES2 | 17.42±0.93ce | 0.25±0.00d | 0.83±0.01h | 70.68±3.98df |
| | TUTVsES3 | 17.57±1.68ce | 0.14±0.00gh | 0.93±0.01fg | 125.05±10.49b |
| | TUTPvES1 | 21.10±0.45a | 0.26±0.00d | 0.97±0.01ef | 81.15±1.58de |
| | TUTPvES2 | 19.38±0.32ac | 0.30±0.00c | 1.17±0.01c | 64.06±1.05ef |
| | TUTPvES3 | 19.10±0.35bd | 0.14±0.01gh | 1.28±0.01b | 136.43±8.93ab |
| | TUTGmGH1 | 13.00±0.53fg | 0.36±0.01b | 1.09±0.00d | 36.11±2.06g |
| | TUTGmGH2 | 11.24±0.29g | 0.46±0.02a | 1.10±0.00d | 24.43±0.23g |
| | TUTGmGH3 | 12.13±0.39fg | 0.15±0.00g | 1.48±0.00a | 80.86±2.33de |
| | UNINOCULATED | 12.11±0.31fg | 0.10±0.00i | 0.61±0.01j | 121.01±2.11bc |
| | ***F-statistics*** | 25.58*** | 253.26** | 326.71** | 47.15*** |
| **Velvet bean** | TUTVuSA1 | 8.01±0.27d | 0.04±0.00h | 1.87±0.01d | 200.25±7.57c |
| cv. IIHR PS 1 | TUTVuSA2 | 8.98±0.36c | 0.06±0.00b | 1.56±0.01h | 149.66±9.70e |
| | TUTVuSA3 | 9.09±0.34c | 0.09±0.00a | 2.44±0.06c | 101.00±3.33gh |
| | TUTMgSA1 | 7.55±0.02de | 0.09±0.00a | 2.72±0.09b | 83.88±0.56h |
| | TUTMgSA2 | 5.62±0.14f | 0.05±0.00de | 1.73±0.01eg | 112.04±5.33gh |
| | TUTMgSA3 | 7.06±0.14e | 0.06±0.00d | 1.79±0.06df | 117.67±2.38ef |
| | TUTVsES1 | 4.09±0.06g | 0.02±0.00i | 1.81±0.04de | 204.05±10.21cd |

| | | | | | |
|---|---|---|---|---|---|
| | TUTVsES2 | 10.55±0.37b | 0.04±0.00h | 1.71±0.00eg | 263.75±10.80a |
| | TUTVsES3 | 5.98±0.07f | 0.05±0.00fg | 1.68±0.01fg | 119.06±1.66fg |
| | TUTPvES1 | 8.72±0.03c | 0.05±0.00g | 1.64±0.04gh | 174.04±1.27d |
| | TUTPvES2 | 12.45±0.35a | 0.05±0.00ef | 2.37±0.03c | 249.00±6.90b |
| | TUTPvES3 | 5.99±0.14f | 0.06±0.00c | 2.87±0.04a | 99.83±0.96h |
| | TUTGmGH1 | 9.35±0.19c | 0.05±0.00ef | 1.75±0.00dg | 187.01±3.53d |
| | TUTGmGH2 | 7.46±0.00de | 0.05±0.00ef | 1.75±0.00dg | 149.02±0.00e |
| | TUTGmGH3 | 7.54±0.00de | 0.06±0.00c | 1.81±0.00de | 125.66±0.00ef |
| | UNINOCULATED | 5.99±0.09f | 0.05±0.00ef | 1.75±0.00dg | 119.08±2.38ef |
| | ***F-statistics*** | 96.27** | 363.13** | 127.96** | 78.76** |
| **Jack bean** | TUTVuSA1 | 8.99±0.09e | 0.04±0.00fg | 0.98±0.01h | 224.75±2.24c |
| cv. Accession 493 | TUTVuSA2 | 5.81±0.17i | 0.03±0.00g | 2.83±0.32d | 193.66±5.11d |
| | TUTVuSA3 | 7.70±0.09g | 0.06±0.01de | 1.97±0.01g | 128.33±18.49e |
| | TUTMgSA1 | 10.86±0.06c | 0.11±0.00bc | 2.88±0.02cd | 98.73±1.33fg |
| | TUTMgSA2 | 12.21±0.16b | 0.14±0.01a | 2.94±0.02c | 87.21±7.45gh |
| | TUTMgSA3 | 14.56±0.09a | 0.07±0.00d | 2.15±0.00f | 208.00±0.55c |
| | TUTVsES1 | 8.79±0.10ef | 0.04±0.00g | 2.25±0.02e | 219.75±1.19c |
| | TUTVsES2 | 7.20±0.16h | 0.06±0.00de | 2.89±0.04cd | 120.00±1.21f |
| | TUTVsES3 | 9.49±0.30d | 0.05±0.00ef | 2.01±0.00g | 189.08±6.51d |
| | TUTPvES1 | 5.71±0.03i | 0.04±0.00g | 2.17±0.01f | 142.75±0.82e |
| | TUTPvES2 | 8.61±0.4f | 0.03±0.00g | 2.32±0.01e | 287.01±9.01a |
| | TUTPvES3 | 5.28±0.14j | 0.02±0.00h | 2.83±0.02d | 264.00±5.54b |
| | TUTGmGH1 | 4.49±0.00k | 0.11±0.00bc | 2.84±0.00d | 40.82±0.49i |
| | TUTGmGH2 | 9.50±0.09d | 0.11±0.00b | 3.11±0.00b | 86.36±0.48h |
| | TUTGmGH3 | 10.83±0.06c | 0.10±0.00c | 3.40±0.08a | 108.30±0.68fg |
| | UNINOCULATED | 5.81±0.11i | 0.03±0.00g | 1.77±0.01g | 193.67±1.01d |
| | ***F-statistics*** | 476.57** | 96.27** | 518.1** | 138.43** |
| **Pigeonpea** | TUTVuSA1 | 14.42±0.06d | 0.11±0.00h | 0.40±0.02fg | 131.09±2.44d |
| ICEAP500557 | TUTVuSA2 | 17.27±0.24c | 0.12±0.00gh | 0.39±0.00fg | 143.92±1.41b |
| | TUTVuSA3 | 14.91±0.23d | 0.09±0.00i | 0.34±0.01g | 165.67±0.58a |
| | TUTMgSA1 | 13.89±0.97d | 0.09±0.00i | 0.43±0.03ef | 154.33±0.09ab |

| | | | | | |
|---|---|---|---|---|---|
| | TUTMgSA2 | 21.47±0.29a | 0.31±0.00b | 0.86±0.04a | 69.25±0.09k |
| | TUTMgSA3 | 18.25±0.01bc | 0.40±0.00a | 0.77±0.06b | 45.62±0.27l |
| | TUTVsES1 | 14.00±0.47d | 0.10±0.00hi | 0.02±0.00h | 140.00±1.09c |
| | TUTVsES2 | 10.87±0.12f | 0.21±0.00e | 0.04±0.00h | 51.76±0.43h |
| | TUTVsES3 | 9.53±0.08g | 0.13±0.00fg | 0.50±0.03de | 73.31±2.63f |
| | TUTPvES1 | 12.05±0.42e | 0.13±0.00fg | 0.63±0.03c | 92.69±1.44f |
| | TUTPvES2 | 17.34±0.03c | 0.24±0.02d | 0.08±0.00h | 72.25±4.81i |
| | TUTPvES3 | 19.01±0.10b | 0.26±0.00c | 0.09±0.00h | 73.16±0.74j |
| | TUTGmGH1 | 7.75±0.37h | 0.10±0.00hi | 0.37±0.03fg | 77.05±0.55j |
| | TUTGmGH2 | 17.13±0.01c | 0.27±0.00c | 0.91±0.02a | 63.44±0.50jk |
| | TUTGmGH3 | 13.93±0.56d | 0.14±0.00f | 0.55±0.02d | 99.05±1.30e |
| | UNINOCULATED | 8.77±0.31h | 0.09±0.00i | 0.04±0.00h | 97.44±2.63f |
| | ***F-statistics*** | 103.53** | 470.27** | 123.98** | 720.27** |
| **Mungbean** | TUTVuSA1 | 14.48±0.00de | 0.22±0.01de | 0.82±0.00e | 65.81±1.84hi |
| cv. VC1973A | TUTVuSA2 | 13.97±0.00df | 0.13±0.00h | 1.35±0.00c | 107.46±2.11d |
| | TUTVuSA3 | 16.24±0.00b | 0.24±0.01cd | 0.27±0.00g | 67.66±1.71gi |
| | TUTMgSA1 | 13.60±0.43ef | 0.30±0.02b | 0.78±0.00e | 45.33±2.79ij |
| | TUTMgSA2 | 15.97±0.48b | 0.18±0.00f | 0.49±0.02f | 88.72±1.63eg |
| | TUTMgSA3 | 12.59±0.70g | 0.33±0.00a | 1.85±0.08a | 38.15±0.64j |
| | TUTVsES1 | 17.84±0.02a | 0.09±0.01jk | 0.14±0.02h | 198.22±14.53b |
| | TUTVsES2 | 18.03±0.09a | 0.07±0.01k | 0.13±0.01h | 257.57±19.46a |
| | TUTVsES3 | 14.60±0.26cd | 0.12±0.00hi | 0.56±0.01f | 121.66±3.60c |
| | TUTPvES1 | 9.89±0.48h | 0.12±0.00h | 1.60±0.08b | 82.42±3.68de |
| | TUTPvES2 | 15.88±0.03b | 0.15±0.01g | 0.56±0.00f | 105.87±4.10d |
| | TUTPvES3 | 17.69±0.08a | 0.10±0.00ij | 0.55±0.00f | 176.09±0.17c |
| | TUTGmGH1 | 15.45±0.06bc | 0.17±0.01fg | 0.47±0.06f | 90.88±3.47ef |
| | TUTGmGH2 | 17.93±0.24a | 0.21±0.00e | 0.50±0.01f | 85.38±0.09fh |
| | TUTGmGH3 | 13.36±0.11fg | 0.25±0.00c | 0.95±0.00d | 53.44±0.31hj |
| | UNINOCULATED | 16.27±0.00b | 0.24±0.00cd | 0.26±0.00g | 67.79±1.11gi |
| | ***F-statistics*** | 61.37** | 162.19** | 229.23** | 79.75** |

Values (Mean ±SE) with dissimilar letters in a column are significant at *p≤0.05, **p≤0.01, ***p≤0.001 and ns=not significant.

Table S2: Photosynthetic rate (A), stomatal conductance (gs), transpiration rate (E) and WUE of native rhizobial isolates at Tshwane University of Technology under glasshouse conditions in 2022. Mean values with dissimilar letters in a column are significantly different at $p<0.05$.

| | **Isolate numbers** | **A** | **gs** | **E** | **WUE** |
|---|---|---|---|---|---|
| | | **μmol $CO_2$ $m^{-2}s^{-1}$** | **mmol $m^{-1}.s^{-1}$** | **mmol $m^{-2}.H_2O$** | **mmol $CO_2$ $m^{-1}$. $H_2O$** |
| **Cowpea** | TUTVuSA1 | 6.93±0.90h | 0.10±0.00d-f | 0.11±0.03c | 69.03±10.12hi |
| cv. IT10K-866-1 | TUTVuSA2 | 7.39±1.22f-h | 0.06±0.01g-j | 0.86±0.16bc | 123.17±6.97ef |
| | TUTVuSA3 | 10.74±1.42b-e | 0.13±0.03bc | 2.26±0.86a | 82.62±16.47gh |
| | TUTMgSA1 | 7.81±1.57f-h | 0.12±0.02cd | 1.61±0.67ab | 65.08±3.88hi |
| | TUTMgSA2 | 8.95±0.66c-g | 0.07±0.00f-h | 0.46±0.17c | 127.86±10.13ef |
| | TUTMgSA3 | 17.19±0.70a | 0.16±0.01a-b | 0.49±0.03c | 107.44±7.23fg |
| | TUTVsES1 | 7.94±0.32e-h | 0.11±0.01c-e | 0.50±0.02c | 89.55±5.57hi |
| | TUTVsES2 | 9.85±1.42c-f | 0.16±0.02ab | 0.41±0.06c | 61.56±8.40hi |
| | TUTVsES3 | 5.57±0.43h | 0.04±0.00i-k | 0.29±0.02c | 139.25±7.90de |
| | TUTPvES1 | 10.01±0.91c-f | 0.05±0.00h-k | 0.30±0.00c | 200.02±9.56bc |
| | TUTPvES2 | 11.62±0.15bc | 0.07±0.00f-h | 0.23±0.00c | 166.00±1.09d |
| | TUTPvES3 | 13.14±0.69b | 0.08±0.01e-g | 0.86±0.31bc | 164.25±2.48d |
| | TUTGmGH1 | 5.47±0.16h | 0.02±0.00k | 0.20±0.00c | 273.05±8.64b |
| | TUTGmGH2 | 8.39±0.62d-g | 0.03±0.00jk | 0.74±0.02c | 279.67±16.21a |
| | TUTGmGH3 | 8.67±0.25d-g | 0.06±0.00g-i | 0.48±0.04c | 144.05±10.09de |
| | COMMERCIAL INOC | 11.06±0.40b-d | 0.09±0.002e-g | 0.40±0.00c | 122.88±2.53ef |
| | NITRATE | 10.86±0.99b-d | 0.06±0.00g-j | 0.75±0.01c | 181.00±4.72c |
| | UNINOCULATED | 8.89±0.22c-g | 0.18±0.00a | 0.44±0.04c | 49.39±2.20i |
| | ***F-statistics*** | 10.93*** | 22.88*** | 3.80*** | 61.99** |
| **Bambara** | TUTVuSA1 | 8.85±0.38c-f | 0.17±0.01a | 0.54±0.08a | 52.06±1.27j |
| **Groundnut** | TUTVuSA2 | 8.96±0.82c-e | 0.05±0.01ij | 0.29±0.16cd | 179.02±9.01b |
| LR. SSD8 | TUTVuSA3 | 9.88±1.04cd | 0.07±0.00f-h | 0.12±0.02e | 141.14±8.55cd |
| | TUTMgSA1 | 9.53±1.18c-e | 0.05±0.00j | 0.48±0.06ab | 190.06±8.04a |
| | TUTMgSA2 | 13.52±2.07a | 0.06±0.01g-i | 0.39±0.08a-c | 225.33±14.10a |
| | TUTMgSA3 | 8.72±0.64c-f | 0.10±0.00cd | 0.49±0.05ab | 87.02±4.49hi |
| | TUTVsES1 | 6.12±0.11fg | 0.12±0.00c | 0.35±0.02bc | 51.00±0.49j |
| | TUTVsES2 | 11.06±0.40a-c | 0.15±0.01b | 0.40±0.00a-c | 73.73±1.96i |

| | | | | | |
|---|---|---|---|---|---|
| | TUTVsES3 | 9.16±0.74c-e | 0.09±0.00de | 0.48±0.04ab | 101.77±7.02gh |
| | TUTPvES1 | 8.63±0.48c-f | 0.11±0.01c | 0.39±0.01a-c | 78.45±2.81i |
| | TUTPvES2 | 5.52±0.59g | 0.05±0.01j | 0.11±0.02e | 110.04±4.97ef |
| | TUTPvES3 | 7.80±0.06d-g | 0.07±0.00f-h | 0.16±0.01de | 111.42±3.86f-g |
| | TUTGmGH1 | 10.21±0.46cd | 0.08±0.00fg | 0.38±0.00a-c | 127.63±8.48c-e |
| | TUTGmGH2 | 6.87±0.05e-g | 0.05±0.00j | 0.11±0.01e | 137.04±4.76c |
| | TUTGmGH3 | 10.53±0.73b-d | 0.08±0.00ef | 0.19±0.01de | 131.63±4.20d-f |
| | COMMERCIAL INOC | 13.03±1.52ab | 0.10±0.01cd | 0.40±0.01a-c | 130.03±1.77d-f |
| | NITRATE | 7.72±0.50d-g | 0.06±0.00h-j | 0.18±0.02de | 128.67±7.23c-e |
| | UNINOCULATED | 9.01±0.08c-e | 0.08±0.00ef | 0.48±0.04ab | 112.63±6.25e-g |
| | ***F-statistics*** | 6.19*** | 53.72** | 7.71*** | 49.92** |
| **Kersting's groundnut** | TUTVuSA1 | 7.30±0.67h | 0.06±0.00h | 0.23±0.19g-j | 121.67±6.27f |
| LR. Dowie | TUTVuSA2 | 17.84±0.48a | 0.12±0.00bc | 0.57±0.01de | 148.67±0.44e |
| | TUTVuSA3 | 7.31±0.09h | 0.12±0.00b | 0.33±0.01e-j | 60.92±0.16i |
| | TUTMgSA1 | 6.88±0.07h | 0.14±0.01a | 0.33±0.01e-j | 49.14±2.01i |
| | TUTMgSA2 | 9.53±0.24ef | 0.06±0.00hi | 0.53±0.00d-f | 158.83±8.28cd |
| | TUTMgSA3 | 14.86±0.93c | 0.14±0.01a | 0.36±0.01d-i | 106.14±2.82gh |
| | TUTVsES1 | 7.20±0.18h | 0.08±0.00ef | 0.49±0.03d-g | 90.00±1.99h |
| | TUTVsES2 | 9.22±0.59e-g | 0.05±0.00hi | 0.14±0.01ij | 184.04±10.53cd |
| | TUTVsES3 | 4.81±0.37i | 0.10±0.00d | 0.07±0.03j | 48.01±3.46i |
| | TUTPvES1 | 12.69±0.70d | 0.07±0.01g | 1.68±0.05a | 181.29±5.53bc |
| | TUTPvES2 | 7.77±0.28g-h | 0.06±0.00gh | 0.15±0.00ij | 129.05±5.50fg |
| | TUTPvES3 | 10.39±0.09e | 0.05±0.00ij | 1.30±0.00b | 207.08±1.73a |
| | TUTGmGH1 | 14.27±0.36c | 0.08±0.00e | 0.47±0.02d-h | 178.38±5.98cd |
| | TUTGmGH2 | 16.45±0.10b | 0.10±0.00d | 0.88±0.30c | 164.05±1.94de |
| | TUTGmGH3 | 12.05±0.33d | 0.06±0.00gh | 0.20±0.01h-j | 200.83±5.70b |
| | COMMERCIAL INOC | 8.11±0.14f-h | 0.04±0.00j | 0.14±0.01ij | 202.75±2.93b |
| | NITRATE | 8.81±0.91fg | 0.07±0.00fg | 0.64±0.01cd | 125.86±11.71f-h |
| | UNINOCULATED | 11.96±0.44d | 0.11±0.00c | 0.26±0.03f-j | 108.73±3.26hi |
| | ***F-statistics*** | 60.23** | 109.39** | 24.37*** | 93.58** |
| **Common bean** | TUTVuSA1 | 5.52±0.25h | 0.06±0.00h | 0.23±0.00h | 92.00±6.76h-k |

| | | | | | |
|---|---|---|---|---|---|
| cv. NUA 721 | TUTVuSA2 | 11.27±0.61de | 0.10±0.01d | 0.39±0.01d-g | 112.07±11.89f-h |
| | TUTVuSA3 | 10.39±0.34ef | 0.08±0.00g | 0.41±0.02d-g | 129.88±7.16ef |
| | TUTMgSA1 | 9.71±0.93fg | 0.05±0.00i | 0.44±0.00d-f | 194.02±18.82c |
| | TUTMgSA2 | 6.48±0.30h | 0.07±0.01g | 0.44±0.01d-g | 92.57±3.67g-k |
| | TUTMgSA3 | 6.41±0.08h | 0.03±0.00j | 0.33±0.06g | 204.67±12.24c |
| | TUTVsES1 | 12.15±0.32cd | 0.09±0.00de | 0.42±0.00d-g | 135.00±4.82ef |
| | TUTVsES2 | 8.44±0.05g | 0.10±0.00d | 0.34±0.02fg | 84.04±0.52i-k |
| | TUTVsES3 | 13.26±0.07c | 0.20±0.00a | 0.48±0.08cd | 66.03±0.09k |
| | TUTPvES1 | 17.56±0.32a | 0.12±0.00c | 0.55±0.00bc | 146.33±2.66de |
| | TUTPvES2 | 15.21±0.25b | 0.17±0.01b | 0.76±0.02a | 89.47±5.18g-j |
| | TUTPvES3 | 12.87±0.44c | 0.08±0.00fg | 0.46±0.01c-e | 158.78±5.46d |
| | TUTGmGH1 | 8.44±0.32g | 0.03±0.00j | 0.63±0.02b | 266.96±10.46a |
| | TUTGmGH2 | 13.09±0.29c | 0.11±0.00c | 0.08±0.01i | 115.64±2.92fg |
| | TUTGmGH3 | 6.52±0.57h | 0.10±0.00de | 0.11±0.01i | 68.20±4.41jk |
| | COMMERCIAL INOC | 12.74±0.58c | 0.06±0.00hi | 0.34±0.02fg | 227.11±5.71b |
| | NITRATE | 9.38±0.34fg | 0.10±0.00d | 0.36±0.02e-g | 91.96±3.11g-j |
| | UNINOCULATED | 9.60±0.86fg | 0.09±0.00ef | 0.60±0.08b | 107.24±12.48f-i |
| | ***F-statistics*** | 53.15** | 178.91** | 25.89*** | 49.19** |
| **Soybean** | TUTVuSA1 | 5.73±0.46i | 0.11±0.00b | 0.03±0.00l | 50.25±3.85k |
| cv. TGX1937-1F | TUTVuSA2 | 5.93±0.31i | 0.08±0.00e | 0.55±0.01e | 71.73±6.02j |
| | TUTVuSA3 | 4.15±0.37i | 0.04±0.00j | 0.12±0.01j | 105.46±9.73fg |
| | TUTMgSA1 | 9.59±0.38ef | 0.08±0.00e | 0.05±0.01l | 121.05±8.48d-f |
| | TUTMgSA2 | 13.09±0.29bc | 0.10±0.00c | 0.09±0.01k | 128.72±2.69de |
| | TUTMgSA3 | 8.73±0.07fg | 0.06±0.00gh | 0.61±0.00d | 141.68±1.33d |
| | TUTVsES1 | 5.73±0.47i | 0.04±0.00j | 0.34±0.02h | 140.37±13.89d |
| | TUTVsES2 | 11.32±0.08d | 0.05±0.00i | 0.63±0.01d | 213.37±5.15c |
| | TUTVsES3 | 9.57±0.15ef | 0.10±0.00 | 0.04±0.00l | 94.09±1.39hi |
| | TUTPvES1 | 7.29±0.32h | 0.10±0.00cd | 0.44±0.00f | 74.99±3.31ij |
| | TUTPvES2 | 13.06±0.79bc | 0.06±0.00hi | 0.36±0.01h | 226.64±10.76bc |
| | TUTPvES3 | 8.34±0.42gh | 0.21±0.00a | 0.74±0.02c | 40.03±2.07k |
| | TUTGmGH1 | 15.73±0.53a | 0.07±0.00g | 0.40±0.00g | 241.07±5.64b |
| | TUTGmGH2 | 9.89±0.43ef | 0.10±0.00cd | 0.91±0.00b | 100.88±3.94fg |

| | | | | | |
|---|---|---|---|---|---|
| | TUTGmGH3 | 13.94±0.45b | 0.04±0.00j | 0.33±0.01h | 313.41±11.02a |
| | COMMERCIAL INOC | 12.56±0.17c | 0.12±0.00b | 0.43±0.02f | 108.60±0.95e-g |
| | NITRATE | 10.07±0.43e | 0.09±0.00d | 1.16±0.02a | 108.35±5.77e-g |
| | UNINOCULATED | 7.18±0.39h | 0.07±0.00f | 0.26±0.00i | 97.62±8.08g |
| | ***F-statistics*** | 66.35** | 510.66** | 879.32** | 110.12** |
| **Velvet bean** | TUTVuSA1 | 7.69±0.21e-g | 0.09±0.00de | 0.23±0.00gh | 88.43±3.91fg |
| cv. IIHR PS 2 | TUTVuSA2 | 10.88±0.21c | 0.10±0.00c | 0.68±0.00d | 107.22±2.09ef |
| | TUTVuSA3 | 8.19±0.09e | 0.16±0.01a | 0.49±0.00d-f | 51.04±1.93gh |
| | TUTMgSA1 | 5.61±0.14ij | 0.04±0.00i | 0.10±0.00h | 132.52±5.26de |
| | TUTMgSA2 | 6.87±0.05f-h | 0.07±0.00fg | 0.18±0.01gh | 104.84±2.04ef |
| | TUTMgSA3 | 11.53±0.19bc | 0.06±0.00gh | 0.36±0.02e-g | 192.20±8.53ab |
| | TUTVsES1 | 7.78±0.47ef | 0.10±0.00c | 0.63±0.06d | 76.43±4.58f-h |
| | TUTVsES2 | 14.02±0.47a | 0.09±0.00d | 0.23±0.02gh | 151.93±0.65cd |
| | TUTVsES3 | 9.71±0.17d | 0.05±0.00hi | 1.20±0.07c | 177.33±5.69bc |
| | TUTPvES1 | 6.63±0.05g-i | 0.16±0.00a | 0.60±0.01de | 42.64±0.5h |
| | TUTPvES2 | 11.62±0.99bc | 0.16±0.00a | 3.04±0.10a | 73.64±8.38f-h |
| | TUTPvES3 | 12.38±0.18b | 0.13±0.00b | 0.28±0.01f-h | 95.95±4.70ef |
| | TUTGmGH1 | 8.53±0.14e | 0.08±0.00e | 1.18±0.09c | 104.05±2.28ef |
| | TUTGmGH2 | 14.34±0.34a | 0.08±0.00e | 0.37±0.28e-g | 173.78±4.10bc |
| | TUTGmGH3 | 10.83±0.35c | 0.05±0.00 | 1.04±0.01c | 222.45±8.48a |
| | NITRATE | 6.55±0.09hi | 0.07±0.00f | 1.87±0.03b | 91.39±2.30f |
| | UNINOCULATED | 4.79±0.60j | 0.03±0.00j | 0.51±0.01d-f | 194.62±46.34ab |
| | ***F-statistics*** | 62.74** | 193.71** | 92.95** | 19.54*** |
| **Jack bean** | TUTVuSA1 | 5.80±0.26e | 0.03±0.00l | 0.65±0.00de | 230.42±10.73a |
| cv. Accession 498 | TUTVuSA2 | 3.55±0.14f | 0.05±0.00j | 1.18±0.01b | 72.60±6.18fg |
| | TUTVuSA3 | 12.12±0.23a | 0.07±0.00h | 0.55±0.01 | 164.10±3.23b |
| | TUTMgSA1 | 8.99±0.46c | 0.22±0.00a | 0.42±0.01 | 41.81±2.02j |
| | TUTMgSA2 | 5.54±0.15e | 0.11±0.00de | 0.26±0.00g | 50.72±1.37ij |
| | TUTMgSA3 | 3.43±0.18fg | 0.04±0.00j | 1.55±0.00a | 80.03±5.32f |
| | TUTVsES1 | 10.42±0.23b | 0.09±0.00fg | 0.28±0.04fg | 116.02±1.66c |
| | TUTVsES2 | 11.89±0.64a | 0.10±0.00de | 0.55±0.00ef | 113.95±4.21c |

| | | | | | |
|---|---|---|---|---|---|
| | TUTVsES3 | 7.10±0.05d | 0.13±0.00c | 0.36±0.15fg | 56.86±2.03i |
| | TUTPvES1 | 5.56±0.01e | 0.09±0.00f | 0.29±0.00fg | 59.58±0.66i |
| | TUTPvES2 | 2.74±0.07g | 0.03±0.00k | 1.44±0.10a | 82.46±0.63ef |
| | TUTPvES3 | 7.09±0.07d | 0.17±0.00b | 0.57±0.01e | 42.46±1.21j |
| | TUTGmGH1 | 10.18±0.34b | 0.11±0.00d | 0.88±0.02c | 92.54±4.29de |
| | TUTGmGH2 | 5.07±0.22e | 0.08±0.00g | 1.05±0.01b | 61.80±1.21gh |
| | TUTGmGH3 | 8.73±0.7c | 0.09±0.00f | 0.63±0.02de | 95.29±0.86d |
| | NITRATE | 12.38±0.18a | 0.10±0.00e | 0.27±0.00fg | 122.07±1.75c |
| | UNINOCULATED | 10.53±0.16b | 0.06±0.00i | 0.76±0.01cd | 164.36±2.20b |
| | ***F-statistics*** | 157.03** | 293.66** | 77.41** | 174.72** |
| **Pigeonpea** cv. ICEAP00850 | TUTVuSA1 | 8.60±0.20i | 0.17±0.01b | 0.53±0.02g | 51.22±0.81i |
| | TUTVuSA2 | 14.22±0.36d | 0.17±0.01b | 0.96±0.00c | 82.61±2.43h |
| | TUTVuSA3 | 10.64±0.07g | 0.10±0.00cd | 0.55±0.00fg | 103.40±0.91fg |
| | TUTMgSA1 | 11.59±0.31f | 0.11±0.00cd | 0.64±0.03ef | 107.83±3.03f |
| | TUTMgSA2 | 8.50±0.08i | 0.06±0.00h | 1.48±0.04b | 147.95±3.98e |
| | TUTMgSA3 | 12.96±0.15e | 0.10±0.00de | 0.52±0.06g | 133.42±0.74e |
| | TUTVsES1 | 7.94±0.58ij | 0.05±0.00h | 0.83±0.01d | 165.78±3.88cd |
| | TUTVsES2 | 16.12±0.42b | 0.11±0.00cd | 0.62±0.06e-g | 149.54±4.13de |
| | TUTVsES3 | 11.98±0.17f | 0.06±0.00h | 0.89±0.05cd | 217.30±10.88a |
| | TUTPvES1 | 10.36±0.17g | 0.10±0.00cd | 0.33±0.02h | 100.93±1.75fg |
| | TUTPvES2 | 14.20±0.18d | 0.08±0.01g | 1.55±0.03b | 185.87±16.99b |
| | TUTPvES3 | 17.61±0.02a | 0.10±0.00de | 0.21±0.00i | 179.92±1.61bc |
| | TUTGmGH1 | 11.97±0.28f | 0.20±0.00a | 0.72±0.01e | 58.77±1.41i |
| | TUTGmGH2 | 9.48±0.20h | 0.11±0.00cd | 1.84±0.6a | 88.62±1.96gh |
| | TUTGmGH3 | 7.27±0.16j | 0.08±0.01fg | 0.86±0.03cd | 88.79±5.94gh |
| | NITRATE | 15.36±0.07c | 0.11±0.00c | 0.96±0.01c | 136.35±0.53e |
| | UNINOCULATED | 10.32±0.25g | 0.09±0.00ef | 0.52±0.03g | 115.08±6.61f |
| | ***F-statistics*** | 137.95** | 147.37** | 179.60** | 64.02** |
| **Mungbean** cv. VC6 153 (B-20P | TUTVuSA1 | 9.19±0.37g | 0.15±0.02a | 0.39±0.02g-i | 61.62±4.18h |
| | TUTVuSA2 | 9.29±0.68g | 0.09±0.00c-e | 0.73±0.02d | 100.65±7.01ef |
| | TUTVuSA3 | 11.23±0.60 | 0.11±0.00b | 0.55±0.01e | 102.26±5.50ef |

| | | | | |
|---|---|---|---|---|
| TUTMgSA1 | 6.62±0.41ij | 0.08±0.00gh | 1.23±0.03b | 87.51±3.72fg |
| TUTMgSA2 | 12.59±0.03d-f | 0.10±0.00b-d | 0.33±0.03i | 123.97±0.30d |
| TUTMgSA3 | 15.06±1.02b | 0.09±0.00c-e | 0.47±0.01e-g | 163.03±8.08bc |
| TUTVsES1 | 11.69±0.31ef | 0.07±0.00h | 0.37±0.05hi | 169.66±8.37b |
| TUTVsES2 | 18.31±0.67a | 0.10±0.00b-d | 0.78±0.05d | 177.22±6.43b |
| TUTVsES3 | 14.39±0.05bc | 0.07±0.00h | 0.94±0.02c | 210.02±3.75a |
| TUTPvES1 | 9.92±0.19fg | 0.10±0.00b-d | 1.67±0.06a | 99.80±3.96e-g |
| TUTPvES2 | 8.72±0.21gh | 0.08±0.00f-h | 0.52±0.04ef | 111.92±3.63de |
| TUTPvES3 | 13.20±0.38cd | 0.10±0.00bc | 0.36±0.01hi | 126.52±3.77d |
| TUTGmGH1 | 7.34±0.55hi | 0.07±0.00h | 1.30±0.00b | 106.75±4.16e |
| TUTGmGH2 | 5.60±0.28j | 0.03±0.00i | 0.09±0.00j | 201.26±7.92a |
| TUTGmGH3 | 9.88±0.04fg | 0.09±0.00d-f | 0.44±0.01f-h | 111.50±7.05de |
| NITRATE | 12.84±0.75de | 0.08±0.00e-g | 0.76±0.02d | 151.20±5.37c |
| UNINOCULATED | 8.44±0.11gh | 0.10±0.00b-d | 0.34±0.02i | 83.48±1.11g |
| ***F-statistics*** | 47.54** | 33.82*** | 204.77** | 61.58** |

Values (Mean ±SE) with dissimilar letters in a column are significant at *p≤0.05, **p≤0.01, ***p≤0.001 and ns=not significant.

Table S3: Accumulation of nutrient elements in the shoots of diverse legume species nodulated by native rhizobial isolates in the glasshouse in 2021.

| | Treatments | Shoot DM | N | P | K | Mg | Cu | Zn | Mn |
|---|---|---|---|---|---|---|---|---|---|
| | | $g.plant^{-1}$ | | | % | | | $mg.kg^{-1}$ | |
| Cowpea | TUTVuSA1 | 3.40±0.20a | 0.84±0.14d | 0.80±0.12b | 2.63±0.35a | 0.37±0.07b | 263.14±34.97cd | 68.90±3.18b | 641.33±76.70cd |
| cv. IT10K-817-3 | TUTVuSA2 | 3.50±0.30a | 1.62±0.23cd | 0.67±0.01c | 1.61±0.09d | 0.19±0.01d | 285.53±5.67bc | 30.59±2.13f | 935.17±15.29b |
| | TUTVuSA3 | 2.43±0.09b | 3.66±0.55a | 0.98±0.00a | 1.99±0.00c | 0.65±0.00a | 302.42±0.00a | 83.26±0.00a | 1123.51±0.00a |
| | TUTPvES3 | 1.13±0.03cd | 1.23±0.20d | 0.57±0.00c | 2.16±0.00b | 0.28±0.00c | 200.46±0.00e | 62.28±0.00c | 1020.14±0.00b |
| | TUTGmGH1 | 0.43±0.03d | 0.94±0.14d | 0.26±0.00f | 1.04±0.00f | 0.23±0.00cd | 242.57±5.30d | 80.91±3.86a | 682.27±0.27c |
| | TUTGmGH3 | 1.43±0.03c | 2.69±0.43b | 0.30±0.01e | 1.35±0.08ef | 0.35±0.02b | 244.58±5.71d | 46.92±2.74e | 656.77±23.59cd |
| | COMMERCIAL INOC | 3.73±0.49a | 2.27±0.32bc | 0.39±0.00d | 1.50±0.03e | 0.28±0.01c | 149.81±5.01f | 31.13±2.08f | 552.88±22.44d |

| | | | | | | | | | |
|---|---|---|---|---|---|---|---|---|---|
| | *F-statistics* | 37.05*** | 10.05*** | 44.57*** | 90.39*** | 36.19*** | 81.39*** | 117.23*** | 74.77*** |
| Bambara groundnut | TUTVsES1 | 3.43±0.49a | 2.99±0.42ab | 0.36±0.01e | 1.66±0.01e | 0.32±0.00c | 110.32±0.00c | 58.79±0.07a | 717.19±1.93c |
| LR.SSD5 | TUTVsES2 | 2.53±0.28bc | 1.85±0.26bc | 0.65±0.01a | 3.18±0.03a | 0.51±0.02a | 130.51±0.02a | 51.26±0.10b | 560.81±3.71d |
| | TUTVsES3 | 3.13±0.54ab | 1.87±0.28bc | 0.52±0.01c | 2.85±0.02b | 0.38±0.00b | 160.38±0.00b | 42.95±0.21c | 457.30±0.04e |
| | TUTMgSA3 | 1.50±0.10cd | 2.33±0.34a-c | 0.59±0.01b | 2.44±0.06c | 0.23±0.00d | 130.23±0.00d | 39.30±0.94d | 483.72±7.30e |
| | TUTGmGH1 | 1.13±0.03d | 1.66±0.23c | 0.47±0.01d | 1.68±0.01e | 0.15±0.01f | 150.15±0.01f | 31.31±0.61e | 394.80±5.02f |
| | TUTGmGH2 | 1.13±0.03d | 1.35±0.21c | 0.45±0.02d | 1.10±0.01g | 0.39±0.00b | 120.39±0.00b | 31.02±0.01e | 233.63±2.31g |
| | TUTGmGH3 | 1.43±0.09cd | 1.36±0.26c | 0.31±0.01f | 1.36±0.03f | 0.15±0.00f | 170.15±0.00f | 30.23±0.64f | 819.14±33.33b |
| | COMMERCIAL INOC | 3.83±0.58a | 3.15±0.78a | 0.45±0.00d | 1.95±0.00d | 0.17±0.00e | 140.17±0.00e | 42.56±0.00c | 1013.45±0.00a |
| | *F-statistics* | 9.44*** | | 252.93** | 194.72** | 369.12** | 53.68*** | 508.88** | 681.87** |
| Kersting's bean | TUTMgSA1 | 1.20±0.75c | 1.54±0.22bc | 0.42±0.02f | 2.40±0.02d | 0.22±0.01d | 301.86±0.86f | 50.75±0.15b | 828.08±7.29c |
| LR.Puffeun | TUTMgSA2 | 1.97±0.09a | 1.00±0.15c | 0.59±0.01c | 1.72±0.01ef | 0.22±0.00d | 332.95±1.33de | 30.76±0.30f | 1218.85±28.13a |
| | TUTMgSA3 | 1.83±0.15b | 2.94±0.42a | 0.72±0.02a | 2.09±0.02e | 0.33±0.00bc | 351.18±1.50d | 40.17±0.05cd | 1130.56±2.06a |
| | TUTVuSA 3 | 1.33±0.15c | 1.29±0.20bc | 0.66±0.00b | 3.24±0.02c | 0.36±0.01b | 415.81±2.30c | 26.22±0.02g | 552.12±1.71de |
| | TUTPvES1 | 0.87±0.09d | 1.52±0.21bc | 0.58±0.01c | 5.02±0.05a | 0.47±0.01a | 279.74±0.67g | 59.82±0.13a | 436.59±1.32fg |
| | TUTPvES3 | 0.97±0.07d | 1.87±0.28b | 0.41±0.01f | 4.53±0.03b | 0.19±0.00e | 324.54±2.41e | 32.79±0.31ef | 618.81±0.74de |
| | TUTGmGH2 | 0.77±0.07d | 0.99±0.14c | 0.48±0.04d | 2.21±0.15f | 0.31±0.03c | 262.02±2.28gh | 41.28±2.99c | 534.81±66.70ef |
| | COMMERCIAL INOC | 1.97±0.78a | 1.50±0.21bc | 0.46±0.00e | 1.89±0.00f | 0.19±0.00e | 519.34±14.41a | 35.52±0.78de | 953.73±10.10b |
| | *F-statistics* | 25.28*** | 7.55*** | 100.16*** | 694.16** | 61.44*** | 179.30** | 68.03*** | 64.13*** |
| Common bean | TUTPvES1 | 4.70±2.60ac | 2.93±0.43a | 0.49±0.00c | 3.92±0.04c | 0.28±0.00c | 700.86±28.13b | 42.99±0.59c | 758.43±6.92b |
| cv. NUA 734 | TUTPvES2 | 3.33±0.12c | 2.23±0.33ab | 0.79±0.01a | 4.29±0.01b | 0.46±0.02a | 379.45±1.55bc | 71.37±0.04a | 547.37±2.02d |
| | TUTPvES3 | 2.77±0.18c | 3.01±0.43a | 0.25±0.01f | 4.29±0.17b | 0.17±0.01d | 235.74±5.88c | 34.67±0.58d | 658.21±1.66c |
| | TUTGmGH2 | 2.43±0.12c | 2.48±0.34ab | 0.33±0.02e | 1.39±0.05f | 0.39±0.01ab | 244.28±5.73c | 48.84±2.96c | 657.74±24.07c |

| | | | | | | | | | |
|---|---|---|---|---|---|---|---|---|---|
| | TUTGmGH3 | 2.27±0.07c | 1.56±0.22b | 0.38±0.01d | 2.79±0.08e | 0.28±0.02c | 276.75±13.47c | 56.25±3.91b | 696.18±13.97bc |
| | COMMERCIAL INOC | 4.30±0.96b | 2.93±0.43a | 0.63±0.00b | 3.36±0.08d | 0.37±0.00b | 1355.65±344.10a | 58.58±1.07b | 940.82±0.17a |
| | NITRATE | 3.23±0.44c | 1.24±0.20b | 0.16±0.01g | 6.63±0.07a | 0.18±0.00d | 408.30±15.85bc | 18.86±0.14e | 217.82±2.21e |
| | *F-statistics* | 11.01*** | 3.15* | 361.75*** | 374.63*** | 24.09*** | 9.13*** | 59.10*** | 59.24*** |
| Soybean | TUTGmGH1 | 3.20±0.78a | 3.69±0.54a | 0.38±0.00c | 1.75±0.02d | 0.36±0.00b | 200.03±1.80de | 30.24±1.04fg | 385.62±10.04bc |
| cv. TGX1740-2F | TUTGmGH2 | 2.47±0.57a | 2.72±0.50a-c | 0.22±0.00g | 3.20±0.04a | 0.27±0.00ef | 1.95±0.01f | 21.79±0.05h | 675.95±0.91a |
| | TUTGmGH3 | 2.53±1.39a | 2.15±0.31b-d | 0.36±0.01d | 1.74±0.03d | 0.23±0.00g | 258.52±3.30b-d | 31.11±0.61ef | 733.26±1.64a |
| | TUTVuSA1 | 1.27±0.07b | 3.17±0.44ab | 0.25±0.01g | 0.89±0.01i | 0.18±0.00h | 369.94±8.40a | 37.55±0.34d | 674.58±7.72a |
| | TUTMgSA3 | 0.77±0.07b | 2.03±0.29b-e | 0.75±0.01a | 2.91±0.08c | 0.47±0.01a | 302.90±1.20a-c | 58.62±0.98c | 483.01±2.69b |
| | TUTVsES1 | 1.73±0.42b | 1.15±0.16de | 0.52±0.00b | 3.02±0.01b | 0.28±0.01de | 255.56±0.67b-d | 67.45±0.33b | 350.74±2.85c |
| | TUTPvES2 | 1.53±0.07b | 2.49±0.36bc | 0.30±0.01f | 1.18±0.00ef | 0.26±0.00f | 266.98±10b-d | 34.70±0.17de | 638.51±40.57a |
| | TUTPvES3 | 0.93±0.15b | 1.64±0.30c-e | 0.34±0.00e | 1.13±0.00f | 0.32±0.00c | 305.15±0.00ab | 110.84±0.00a | 658.91±0.00a |
| | COMMERCIAL INOC | 3.17±2.32a | 1.26±0.18e | 0.38±0.00c | 1.48±0.01e | 0.27±0.01ef | 162.72±14.97e | 26.93±3.41g | 454.05±106.93bc |
| | *F-statistics* | 7.44*** | 5.99*** | 820.02** | 1220.62** | 247.14** | 18.66*** | 549.20** | 29.73*** |
| Winged bean | TUTMgSA2 | 1.43±0.09c | 1.72±0.24c | 0.51±0.01b | 2.12±0.07b | 0.48±0.01b | 529.66±31.80b | 51.96±0.16c | 1365.55±87.08bc |
| cv.VRWB 4A | TUTVsES1 | 2.03±0.12b | 2.42±0.34a-c | 0.53±0.01b | 2.09±0.06b | 0.49±0.01b | 526.62±33.42b | 51.86±2.69c | 1362.07±88.88bc |
| | TUTPvES3 | 1.90±0.12b | 2.26±0.32a-c | 0.53±0.02b | 2.16±0.04b | 0.43±0.03c | 460.62±18.06b | 73.88±1.23a | 1307.97±41.74d |
| | TUTGmGH1 | 2.93±0.17a | 3.24±0.47ab | 0.55±0.01b | 1.85±0.04c | 0.41±0.01c | 487.31±1.11b | 57.01±2.47bc | 1508.94±4.70b |
| | TUTGmGH2 | 2.03±0.12b | 3.39±0.54a | 0.73±0.00a | 2.36±0.02a | 0.54±0.00a | 748.53±21.76a | 63.10±3.18b | 1979.70±38.76a |
| | TUTGmGH3 | 1.77±0.18bc | 1.99±0.28bc | 0.43±0.00c | 1.27±0.01d | 0.33±0.00d | 562.77±59.53b | 42.88±0.10d | 1525.51±26.19b |
| | *F-statistics* | 13.86*** | 3.16* | 61.37*** | 69.31*** | 27.86*** | 9.72*** | 27.53*** | 18.80*** |
| Velvet bean | TUTVuSA1 | 2.67±0.12de | 1.33±0.19a | 0.46±0.03c | 1.16±0.02d | 0.26±0.00b | 554.54±2.98c | 36.65±0.95g | 695.42±48.79d-f |
| cv. IIHR PS 1 | TUTVuSA2 | 4.43±0.20a | 1.21±0.17a | 0.30±0.00g | 1.16±0.03d | 0.17±0.00g | 537.82±21.58c | 37.51±0.32g | 748.50±12.20c-e |

| | | | | | | | | | |
|---|---|---|---|---|---|---|---|---|---|
| | TUTVuSA3 | 4.33±0.23a | 1.36±0.20a | 0.40±0.01de | 1.54±0.01bc | 0.22±0.00de | 483.43±16.84 | 59.16±0.18c-e | 624.33±5.75fg |
| | TUTMgSA1 | 3.30±0.15bc | 1.27±0.18a | 0.34±0.02fg | 1.38±0.13bd | 0.20±0.02d-g | 561.96±20.03c | 46.69±4.44f | 786.78±16.99b-e |
| | TUTMgSA2 | 3.43±0.07bc | 1.38±0.20a | 0.35±0.01fg | 1.48±0.07bd | 0.21±0.00d-f | 537.27±2.20c | 55.63±0.51e | 680.26±29.67ef |
| | TUTMgSA3 | 2.67±0.09de | 1.17±0.17a | 0.41±0.00de | 1.45±0.02bd | 0.23±0.02cd | 696.35±31.24b | 74.67±2.42a | 870.89±77.37ab |
| | TUTVsES1 | 3.10±0.17b-d | 1.38±0.20a | 0.42±0.02c-e | 1.48±0.09bd | 0.25±0.01bc | 708.46±58.67b | 63.68±5.99cd | 759.08±21.30b-e |
| | TUTVsES2 | 3.03±0.18cd | 1.19±0.17a | 0.33±0.02fg | 1.21±0.05cd | 0.18±0.02fg | 455.31±2.73c | 43.10±1.38ef | 609.33±12.08fg |
| | TUTVsES3 | 2.33±0.19e | 1.23±0.18a | 0.45±0.00cd | 1.62±0.02b | 0.26±0.01b | 864.10±19.40a | 56.40±2.35de | 803.02±7.48bd |
| | TUTPvES2 | 3.63±0.15b | 1.30±0.19a | 0.59±0.02a | 2.55±0.00a | 0.33±0.01a | 309.03±1.28d | 62.12±0.00c-e | 555.50±9.50g |
| | TUTPvES3 | 4.23±0.20a | 1.22±0.17a | 0.51±0.00b | 1.62±0.13b | 0.31±0.00b | 889.18±93.03a | 71.46±2.57ab | 770.04±90.90b-e |
| | TUTGmGH1 | 3.60±0.17b | 1.32±0.19a | 0.37±0.03ef | 1.56±0.01bc | 0.28±0.00b | 488.01±24.79c | 65.95±0.90bc | 858.50±4.11a-c |
| | TUTGmGH2 | 3.50±0.12bc | 1.22±0.17a | 0.41±0.01de | 1.46±0.02bd | 0.21±0.00d-f | 728.79±14.98b | 76.69±0.41a | 951.67±11.19a |
| | TUTGmGH3 | 2.68±0.11de | 1.22±0.17a | 0.30±0.01g | 1.37±0.34bd | 0.19±0.01e-g | 745.57±1.94b | 76.57±0.35a | 962.37±3.20a |
| | *F-statistics* | 16.21*** | 0.16ns | 24.77*** | 9.59*** | 24.99*** | 24.61*** | 34.39*** | 11.17*** |
| Jack bean | TUTVuSA2 | 4.60±0.29a | 1.25±0.21a | 0.42±0.01a | 1.48±0.06a | 0.26±0.00b | 1071.56±17.07a | 35.52±0.51d | 550.51±11.34d |
| cv. Accession 493 | TUTMgSA3 | 2.80±0.10de | 1.44±0.21a | 0.32±0.02bc | 1.36±0.03ab | 0.23±0.00c | 680.28±46.70c | 45.67±0.85c | 666.30±14.78b |
| | TUTVsES1 | 2.53±0.09e | 1.58±00.22a | 0.31±0.00bc | 1.28±0.02b | 0.28±0.00a | 910.45±11.22b | 56.70±6.03b | 577.12±21.23cd |
| | TUTVsES2 | 3.53±0.09b | 1.51±0.22a | 0.34±0.02b | 1.41±0.08ab | 0.23±0.00cd | 879.03±7.32b | 69.11±0.32a | 622.97±3.96bc |
| | TUTPvES2 | 2.00±0.10f | 1.59±0.27a | 0.29±0.01c | 1.34±0.01b | 0.24±0.01c | 842.34±39.74b | 43.56±0.35c | 553.54±16.24d |
| | TUTVsES3 | 3.23±0.09bc | 1.62±0.22a | 0.28±0.00c | 1.31±0.01b | 0.21±0.00d | 610.11±17.24c | 70.13±0.09a | 957.33±31.15a |
| | *F-statistics* | 38.61*** | 0.40ns | 14.54*** | 2.81* | 26.13*** | 36.60*** | 32.35*** | 70.22*** |
| PigeonPea | TUTVuSA1 | 0.87±0.03b | 1.12±0.16b | 0.68±0.01a | 1.31±0.01b | 0.43±0.00a | 1071.56±17.07c | 35.52±0.51g | 550.51±11.34e |
| cv. ICEAP500557 | TUTVuSA2 | 0.97±0.09ab | 1.19±0.23b | 0.29±0.01e | 0.57±0.01c | 0.19±0.00c | 1459.07±3.92b | 57.43±0.25d | 1627.03±9.43a |
| | TUTPvES2 | 0.77±0.09b | 1.32±0.19b | 0.55±0.00bc | 1.25±0.01b | 0.34±0.01ab | 1597.68±53.90a | 87.27±0.58a | 1045.21±18.03c |
| | TUTGmGH1 | 0.97±0.09ab | 1.26±0.19b | 0.46±0.01d | 1.43±0.00b | 0.23±0.00bc | 576.99±45.45e | 42.19±0.33f | 591.41±36.29e |

| | | | | | | | | | |
|---|---|---|---|---|---|---|---|---|---|
| | TUTGmGH2 | 1.17±0.07a | 1.53±0.21b | 0.50±0.03cd | 1.75±0.20a | 0.36±0.10ab | 651.16±26.95de | 62.27±1.81c | 1378.66±35.78b |
| | TUTGmGH3 | 0.70±0.10b | 2.47±0.35a | 0.60±0.01b | 1.26±0.04b | 0.30±0.00bc | 1581.54±24.29ab | 73.52±0.17b | 826.40±13.84d |
| | *F-statistics* | 4.28* | 4.81* | 32.47*** | 19.70*** | 4.57** | 119.46*** | 265.80*** | 255.10*** |
| Mungbean | TUTVuSA2 | 0.90±0.12b | 1.49±0.23b | 0.69±0.00a | 2.58±0.00a | 0.59±0.00a | 510.83±0.00b | 37.63±0.00bc | 1100.03±0.00b |
| cv. VC1973A | TUTMgSA2 | 0.90±0.00b | 1.26±0.20b | 0.41±0.01bc | 1.46±0.02c | 0.21±0.00bc | 728.79±14.98a | 76.69±0.41a | 951.67±11.19bc |
| | TUTMgSA3 | 0.77±0.09b | 1.52±0.22b | 0.50±0.03b | 1.80±0.16bc | 0.50±0.05a | 600.32±67.95b | 42.03±3.30b | 1503.11±111.29a |
| | TUTPvES 1 | 0.87±0.09b | 1.42±0.21b | 0.39±0.09bc | 1.78±0.22bc | 0.29±0.11bc | 282.87±65.67c | 34.98±0.62c | 773.73±211.48cd |
| | TUTGmGH2 | 1.17±0.03a | 3.36±0.51a | 0.47±0.00b | 1.87±0.03b | 0.34±0.01b | 518.83±0.20b | 37.15±0.41c | 1144.80±2.67b |
| | TUTGmGH3 | 0.90±0.00b | 1.42±0.21b | 0.30±0.00c | 1.56±0.00bc | 0.18±0.00c | 223.17±10.65c | 21.95±1.22d | 569.83±14.44d |
| | *F-statistics* | 3.67* | 8.52** | 12.39*** | 12.29*** | 10.98*** | 23.78*** | 155.83*** | 10.91*** |

Values (Mean ±SE) with dissimilar letters in a column are significant at *p≤0.05, **p≤0.01, ***p≤0.001 and ns=not significant.

Table S4: Accumulation of nutrient elements in the shoots of diverse legume species nodulated by native rhizobial isolates in the glasshouse in 2022.

| | Treatments | Shoot DM | N | P | K | Mg | Cu | Zn | Mn |
|---|---|---|---|---|---|---|---|---|---|
| | | g.plant$^{-1}$ | | | % | | | mg.kg$^{-1}$ | |
| Cowpea | TUTVuSA1 | 3.93±0.68a | 0.71±0.04h | 0.43±0.02ab | 1.62±0.13bc | 0.68±0.00a | 889.18±93.03a | 74.59±2.93a | 1212.93±0.06a |
| cv. IT10K-866-1 | TUTVuSA2 | 3.60±0.21b | 0.98±0.06fg | 0.45±0.01a | 1.94±0.21ab | 0.66±0.02a | 786.63±0.06b | 71.46±2.57ab | 1046.53±0.97b |
| | TUTVuSA3 | 2.93±0.43c | 2.02±0.01d | 0.40±0.03bc | 1.99±0.19a | 0.60±0.03b | 537.82±21.58b | 72.13±0.60ab | 962.63±18.44bc |
| | TUTGmGH1 | 2.03±0.07de | 2.56±0.03b | 0.30±0.00f | 1.14±0.05d | 0.30±0.00d | 230.20±1.20ef | 60.83±03.99cd | 770.09±88.00d |
| | TUTGmGH2 | 0.63±0.09h | 1.99±0.08d | 0.35±0.01c-e | 1.14±0.08d | 0.58±0.00bc | 288.68±4.50de | 46.77±3.24e | 730.39±14.52e |
| | TUTGmGH3 | 0.43±0.07h | 2.20±0.03c | 0.30±0.00ef | 1.16±0.03d | 0.29±0.00d | 229.20±1.24ef | 65.14±0.70bc | 748.50±12.20ef |
| | TUTVsES1 | 0.87±0.03gh | 0.82±0.08gh | 0.33±0.00d-f | 1.32±0.01cd | 0.56±0.01c | 186.95±0.97f | 59.88±1.01cd | 770.04±90.90d |
| | TUTPvES3 | 1.10±0.10fh | 1.34±0.08e | 0.36±0.01cd | 1.25±0.01d | 0.17±0.00e | 261.43±16.59d-f | 54.73±0.24d | 657.80±13.74f |

| | | | | | | | | | |
|---|---|---|---|---|---|---|---|---|---|
| | COMMERCIAL INOC | 2.70±0.12cd | 3.18±0.01a | 0.37±0.03cd | 1.34±0.09cd | 0.62±0.01b | 345.82±0.65d | 60.81±4.86cd | 890.01±1.30cd |
| | *F-statistics* | 71.80*** | 216.17** | 29.50*** | 15.37*** | 214.91** | 71.70*** | 50.15*** | 30.38*** |
| | | | | | | | | | |
| Bambara groundnut | TUTVsES1 | 3.83±0.43a | 2.26±0.34ab | 0.44±0.02a | 1.82±0.02b | 0.26±0.02b | 346.94±2.95a | 40.51±1.88b | 911.21±25.57a |
| LR. SSD8 | TUTVsES2 | 3.97±0.13a | 2.06±0.31b | 0.47±0.01a | 1.93±0.09b | 0.30±0.01a | 276.38±4.25c | 50.98±0.56a | 764.83±3.20b |
| | TUTVsES3 | 2.50±0.31c | 2.95±0.42a | 0.40±0.03b | 2.40±0.04a | 0.25±0.01bc | 315.52±8.24b | 48.16±1.09a | 619.45±1.45c |
| | TUTMgSA3 | 0.83±0.03e | 1.88±0.34b | 0.31±0.00e | 1.47±0.03de | 0.20±0.01de | 231.51±7.83d | 33.28±0.72cd | 569.60±14.63d |
| | TUTPvES2 | 0.73±0.03e | 1.29±0.21b | 0.26±0.01f | 1.29±0.04fg | 0.22±0.00cd | 201.93±3.58 | 31.19±0.46d | 474.85±7.63e |
| | TUTGmGH1 | 3.07±0.09bc | 1.41±0.19b | 0.33±0.03de | 1.19±0.05g | 0.18±0.01e | 204.89±7.64e | 31.29±0.91d | 486.09±14.66ef |
| | TUTGmGH2 | 2.53±0.32c | 1.36±0.20b | 0.34±0.00c-e | 1.36±0.01ef | 0.23±0.01b-d | 241.80±2.22d | 35.87±0.52c | 570.74±2.32d |
| | TUTGmGH3 | 2.63±0.07bc | 1.84±0.25b | 0.36±0.02b-d | 1.49±0.04d | 0.23±0.00b-d | 211.10±2.14e | 34.18±0.82cd | 496.36±0.85e |
| | COMMERCIAL INOC | 3.87±0.09a | 1.89±0.27b | 0.37±0.00bc | 1.65±0.03c | 0.23±0.01cd | 281.67±7.51c | 36.10±0.59c | 614.89±12.41c |
| | *F-statistics* | 28.93*** | 2.79* | 20.96*** | 96.08*** | 17.56*** | 79.24*** | 62.09*** | 152.49** |
| | | | | | | | | | |
| Kersting's groundnut | TUTMgSA1 | 3.20±0.10a | 1.51±0.22b-d | 0.36±0.02a | 1.38±0.07a | 0.36±0.01b | 316.23±1.86a | 40.25±0.68a | 731.84±5.41bc |
| LR. Dowie | TUTMgSA2 | 2.93±0.15a | 1.07±0.15d | 0.35±0.00a | 1.39±0.01a | 0.40±0.00a | 321.60±5.81a | 32.99±0.3c | 662.27±3.44d |
| | TUTMgSA3 | 2.10±0.17b | 2.97±0.42a | 0.29±0.00b | 1.18±0.04b | 0.28±0.01c | 309.48±2.10a | 35.99±0.95b | 872.80±14.47a |
| | TUTVuSA3 | 0.97±0.09h-j | 1.14±0.16cd | 0.25±0.03cd | 0.97±0.02d | 0.21±0.00e | 227.04±2.68d | 20.44±1.18f | 597.47±5.19e |
| | TUTPvES1 | 1.00±0.06h-j | 1.87±0.28bc | 0.21±0.00e | 1.18±0.01b | 0.21±0.00ef | 284.28±4.11b | 27.10±0.01d | 567.52±1.19e |
| | TUTPvES3 | 0.87±0.09h-j | 1.99±0.30b | 0.23±0.01de | 1.06±0.01c | 0.20±0.01ef | 234.31±4.05d | 24.42±0.77de | 481.81±4.62g |
| | TUTGmGH2 | 1.43±0.15d-f | 0.95±0.13d | 0.31±0.00b | 1.05±0.02cd | 0.24±0.01d | 285.08±5.68b | 25.92±1.99d | 716.84±24.96c |
| | TUTGmGH3 | 1.50±0.10c-e | 1.52±0.21b-d | 0.23±0.01de | 0.99±0.01cd | 0.20±0.00ef | 238.36±7.53d | 22.94±1.22ef | 574.90±1.98e |
| | COMMERCIAL INOC | 1.70±0.10cd | 1.12±0.16cd | 0.28±0.00bc | 1.27±0.03b | 0.25±0.01d | 265.10±2.24c | 27.47±0.79d | 751.39±10.74b |
| | *F-statistics* | 49.86*** | 7.06*** | 32.89*** | 45.43*** | 65.83*** | 117.71*** | 66.27*** | 194.31** |
| | | | | | | | | | |
| Common bean | TUTPvES1 | 3.10±0.12ab | 2.78±0.40b | 0.38±0.01ab | 2.35±0.05ab | 0.45±0.01ab | 373.50±3.80a | 55.79±1.32c | 506.36±2.95c |

| | | | | | | | | | |
|---|---|---|---|---|---|---|---|---|---|
| cv. NUA 721 | TUTPvES2 | 2.80±0.17bc | 2.47±0.35a-c | 0.40±0.03a | 2.58±0.08a | 0.43±0.01b | 367.26±3.10a | 98.63±0.16a | 579.27±25.43b |
| | TUTPvES3 | 3.03±0.12ab | 3.22±0.46a | 0.37±0.00ab | 2.25±0.01bc | 0.42±0.01b | 282.99±5.93b | 60.80±0.05b | 795.77±33.58a |
| | TUTGmGH2 | 2.20±0.21de | 1.51±0.21c | 0.33±0.00bc | 1.71±0.20d | 0.30±0.01d | 263.50±28.16bc | 52.52±2.54c | 474.80±4.40cd |
| | TUTGmGH3 | 2.10±0.15df | 2.12±0.30b | 0.30±0.03c | 1.63±0.02d | 0.34±0.00c | 272.03±15.22bc | 34.95±1.58d | 428.42±5.55e |
| | COMMERCIAL INOC | 3.30±0.21a | 3.02±0.42a | 0.33±0.02bc | 2.03±0.03c | 0.47±0.01a | 241.91±1.85c | 57.07±3.27bc | 476.28±6.33cd |
| | *F-statistics* | 20.62*** | 6.01** | 4.80** | 37.33*** | 37.81*** | 24.85*** | 189.34*** | 77.05*** |
| Soybean | TUTGmGH1 | 2.47±0.15ab | 3.82±0.55a | 0.38±0.00b | 1.27±0.00bc | 0.49±0.01b | 509.25±4.62a | 31.77±0.50b-d | 645.91±11.71c |
| cv. TGX1937-1F | TUTGmGH2 | 2.80±0.15a | 2.39±0.34b-e | 0.42±0.01a | 1.55±0.02a | 0.57±0.01a | 442.26±3.40b | 42.73±1.24a | 741.30±34.96b |
| | TUTGmGH3 | 2.07±0.22bc | 2.04±0.28d-f | 0.35±0.01bc | 1.29±0.01bc | 0.53±0.03ab | 392.26±2.37c | 45.92±4.89a | 876.39±9.29a |
| | TUTVuSA1 | 1.97±0.09c | 3.55±0.52ab | 0.25±0.01g | 1.20±0.12cd | 0.36±0.01d | 326.24±14.40e | 27.23±0.78d | 602.29±1.03d |
| | TUTMgSA3 | 1.23±0.09d-f | 1.20±0.17ef | 0.25±0.00g | 1.15±0.02c-e | 0.42±0.00c | 357.66±1.72d | 27.98±0.13d | 657.96±4.79c |
| | TUTVsES2 | 1.43±0.09d | 1.19±0.17ef | 0.33±0.01cd | 1.25±0.06bc | 0.37±0.02d | 306.97±4.14f | 34.55±1.20bc | 308.36±14.06g |
| | TUTPvES2 | 1.37±0.09de | 3.31±0.50a-c | 0.29±0.01f | 1.03±0.03ef | 0.43±0.01c | 322.48±2.45e | 30.88±0.23cd | 410.59±6.78f |
| | TUTPvES3 | 0.93±0.15e | 2.81±0.46a-d | 0.31±0.00de | 1.06±0.02d-f | 0.34±0.00e | 288.02±5.80f | 44.47±2.96a | 548.47±8.68e |
| | COMMERCIAL INOC | 1.93±0.09c | 1.12±0.16f | 0.35±0.01bc | 1.39±0.01b | 0.44±0.00c | 409.57±17.44c | 36.86±0.81b | 592.99±2.99d |
| | *F-statistics* | 16.72*** | 7.43*** | 34.22*** | 13.91*** | 35.83*** | 96.67*** | 72.67*** | 202.71** |
| Velvet bean | TUTVuSA1 | 3.77±0.20ab | 1.29±0.18a | 0.35±0.01b | 1.21±0.01d-f | 0.30±0.00c-e | 540.47±41.04de | 50.00±2.34f | 585.92±19.53b-d |
| cv. IIHR PS 2 | TUTVuSA2 | 2.73±0.09e | 1.15±0.18a | 0.59±0.02b | 2.55±0.00a | 0.33±0.01ab | 924.73±26.24a | 62.12±0.00a | 555.50±9.50d-f |
| | TUTVuSA3 | 4.00±0.21a | 1.29±0.19a | 0.33±0.00b | 1.08±0.01gh | 0.30±0.00c-e | 530.31±21ef | 54.94±0.55c-d | 513.31±20.63gh |
| | TUTMgSA1 | 3.37±0.12b-d | 1.23±0.18a | 0.33±0.00b | 1.25±0.03c-e | 0.30±0.01c-e | 744.97±3.71c | 46.76±1.06g | 534.73±1.58e-g |
| | TUTMgSA2 | 2.97±0.29c-e | 1.30±0.19a | 0.32±0.02b | 1.36±0.03bc | 0.28±0.00ef | 680.28±46.70c | 45.67±0.85g | 666.30±14.78a |
| | TUTMgSA3 | 3.40±0.17b-d | 1.26±0.18a | 0.30±0.00b | 1.21±0.02d-f | 0.32±0.01a-c | 742.41±5.07c | 57.85±1.72bc | 613.90±3.52b |
| | TUTVsES1 | 2.90±0.12de | 1.41±0.20a | 0.38±0.01b | 1.28±0.00cd | 0.30±0.00c-e | 838.15±18.18b | 54.69±0.99c-d | 656.52±8.06a |
| | TUTVsES2 | 2.20±0.20f | 1.54±0.21a | 0.40±0.01b | 1.43±0.02b | 0.32±0.00a-c | 567.52±2.62de | 55.31±0.35c-d | 528.44±1.06fg |

| | | | | | | | | | |
|---|---|---|---|---|---|---|---|---|---|
| | TUTPvES2 | 3.43±0.07bc | 1.20±0.20a | 0.35±0.01b | 1.11±0.02f | 0.34±0.00a | 893.93±7.72ab | 44.98±0.21gh | 601.27±10.26bc |
| | TUTPvES3 | 3.60±0.17ab | 1.35±0.19a | 12.58±12.21a | 1.31±0.03b-d | 0.31±0.00b-d | 609.95±38.90d | 55.95±0.60cd | 565.86±14.87c-e |
| | *F-statistics* | 22.32*** | 0.39ns | 1.00ns | 86.77** | 5.71*** | 66.30** | 34.70*** | 47.78*** |
| Jack bean | TUTVuSA2 | 3.37±0.09e | 1.23±0.17b | 0.68±0.01a | 1.31±0.01b | 0.43±0.00b | 1581.54±24.29a | 57.43±0.25c | 951.67±11.19b |
| cv. Accession 498 | TUTMgSA3 | 4.33±0.15b | 1.61±0.23b | 0.60±0.01b | 1.26±0.04b | 0.21±0.00e | 884.74±38.33d | 37.15±0.04e | 550.51±11.34d |
| | TUTVsES1 | 4.47±0.18a | 1.60±0.23b | 0.41±0.01d | 1.46±0.02b | 0.30±0.00cd | 728.79±14.99e | 53.38±2.05c | 826.40±13.84c |
| | TUTVsES2 | 3.67±0.09d | 1.65±0.24b | 0.61±0.05b | 1.80±0.16a | 0.50±0.05a | 1459.09±3.92b | 73.52±0.17a | 1046.45±2.29a |
| | TUTVsES3 | 3.93±0.19c | 1.65±0.24b | 0.50±0.03c | 1.48±0.08b | 0.25±0.01de | 720.01±73.52e | 42.03±3.30d | 514.10±43.53d |
| | TUTGmGH3 | 4.10±0.00bc | 3.43±0.54a | 0.47±0.00cd | 1.87±0.03a | 0.34±0.01c | 1071.56±17.07c | 66.69±0.41b | 1018.13±0.66a |
| | *F-statistics* | 17.97*** | 5.79** | 41.99*** | 32.77*** | 33.51*** | 181.23*** | 83.88*** | 192.14*** |
| Pigeonpea | TUTVuSA1 | 0.83±0.07c | 2.04±0.29ab | 0.58±0.01a | 5.02±0.05a | 0.22±0.00d | 582.72±2.44c | 46.04±1.18b | 426.06±3.32c |
| cv. ICEAP00850 | TUTVsES1 | 1.27±0.07a | 1.22±0.21bc | 0.38±0.00c | 2.12±0.03b | 0.47±0.01a | 284.99±4.06e | 59.82±0.13a | 436.59±1.32c |
| | TUTMgSA1 | 1.20±0.06a | 1.53±0.21a-c | 0.38±0.00c | 1.11±0.01ef | 0.23±0.01bc | 428.63±1.60d | 42.85±0.90b | 446.61±3.05c |
| | TUTPvES2 | 1.27±0.03a | 1.69±0.24a-c | 0.52±0.00b | 1.50±0.01c | 0.24±0.01bc | 247.40±4.31f | 37.49±1.35c | 808.25±16.07a |
| | TUTGmGH2 | 1.27±0.17a | 1.64±0.23a-c | 0.36±0.01d | 1.23±0.01de | 0.24±0.01bc | 632.66±2.64b | 46.60±1.02b | 586.15±3.27b |
| | TUTGmGH3 | 0.97±0..09bc | 2.34±0.35a | 0.50±0.01b | 1.31±0.04d | 0.26±0.01b | 694.46±1.93a | 38.48±1.85c | 480.52±2.03c |
| | *F-statistics* | 9.16*** | 3.06* | 298.50*** | 1386.26** | 79.12*** | 716.36** | 84.08*** | 50.85*** |
| Mungbean | TUTMgSA2 | 0.77±0.03c | 1.34±0.19cd | 0.42±0.02c | 2.40±0.02d | 0.47±0.01a | 301.86±0.86d | 50.75±0.15c | 828.08±7.29b |
| cv. VC6 153 (B-20P) | TUTMgSA3 | 0.90±0.00c | 2.25±0.32a-c | 0.75±0.01a | 2.91±0.08b | 0.32±0.00d | 305.15±0.00d | 110.84±0.00a | 658.91±0.00c |
| | TUTPvES1 | 1.17±0.12a | 1.52±0.22b-d | 0.41±0.01c | 2.53±0.03c | 0.19±0.00f | 324.54±2.41c | 32.79±0.31d | 618.81±0.74d |
| | TUTGmGH2 | 1.33±0.09a | 1.55±0.25b-d | 0.29±0.01e | 1.70±0.02e | 0.40±0.01b | 468.93±7.03a | 58.62±0.00b | 1017.23±3.06a |
| | TUTGmGH3 | 1.33±0.03a | 2.77±0.49a | 0.66±0.00b | 3.24±0.02a | 0.36±0.01c | 415.81±2.30b | 26.22±0.02e | 552.12±1.71e |
| | *F-statistics* | 11.53*** | 4.46** | 227.02*** | 485.64*** | 134.37*** | 530.24*** | 5313.2** | 2948.1** |

Values (Mean ±SE) with dissimilar letters in a column are significant at *$p \leq 0.05$, **$p \leq 0.01$, ***$p \leq 0.001$ and ns=not significant.

Table S5: N-free plant nutrient solution used in this study

| Stock solution | Molecular form | Source/ Element | Mass/ Litre | Concentration |
|---|---|---|---|---|
| A | $CaCl.2H_2O$ | Ca | 294.1 | 2.0 M |
| B | $KH_2PO_4$ | P | 136.1 | 1.0 M |
| C | Fe-citrate | Fe | 607 | 20.0 mM |
| | $MgSO_4.7H_2O$ | Mg | 123.3 | 0.5 M |
| | $K_2SO_4$ | K | 87.0 | 0.5 M |
| | $MnSO_4.H_2O$ | Mn | 0.338 | 2.0 mM |
| D | $H_3BO_3$ | B | 0.247 | 4.0 mM |
| | $ZnSO_4.7H_2O$ | Zn | 0.288 | 1.0 mM |
| | $CuSO_4.5H_2O$ | Cu | 0.100 | 0.4 mM |
| | $CoSO_4.7H_2O$ | Co | 0.056 | 0.2 mM |
| | $NaMoO_2.H_2O$ | Mo | 0.048 | 0.2 mM |

* For each 10 litres of culture solution, 5.0 ml of each solution (A, B, C and D) was diluted sufficient distilled water to bring the total volume to 10 l
